# Development of novel mitochondrial pyruvate carrier inhibitors for breast cancer treatment

**DOI:** 10.1016/j.jbc.2025.110486

**Published:** 2025-07-16

**Authors:** Tanner J. Schumacher, Zachary S. Gardner, Jon Rumbley, Conor T. Ronayne, Venkatram R. Mereddy

**Affiliations:** 1 Integrated Biosciences Graduate Program, University of Minnesota, Duluth, Minnesota, USA; 2 Department of Pharmacy Practice & Pharmaceutical Sciences, University of Minnesota, Duluth, Minnesota, USA; 3 Department of Chemistry and Biochemistry, University of Minnesota Duluth, Duluth, Minnesota, USA

**Keywords:** mitochondrial pyruvate carrier, aminocarboxycoumarin, tumor metabolism, breast cancer, medicinal chemistry

## Abstract

Reprogrammed metabolism of cancer cells offers a unique target for pharmacological intervention. The mitochondrial pyruvate (Pyr) carrier (MPC) plays important roles in cancer progression by transporting cytosolic Pyr into the mitochondria for use in the tricarboxylic acid cycle. In the current study, a series of novel fluoro-substituted aminocarboxycoumarin derivatives have been evaluated for their MPC inhibition properties. Our studies indicate that the aminocarboxycoumarin template elicits potent MPC inhibitory characteristics, and specifically, structure–activity relationship studies show that the N-methyl-N-benzyl structural template provides the optimal inhibitory capacity. Further respiratory experiments demonstrate that candidate compounds specifically inhibit Pyr-driven respiration without substantially affecting other metabolic fuels, consistent with MPC inhibition. Further, computational inhibitor docking studies illustrate that aminocarboxycoumarin-binding characteristics are nearly identical to that of classical MPC inhibitor UK5099 bound to human MPC, recently determined by cryo-EM. The lead candidate C5 elicits cancer cell proliferation inhibition specifically in monocarboxylate transporter 1–expressing murine breast cancer cells 4T1 and 67NR, consistent with its ability to accumulate intracellular lactate. In vivo tumor growth studies illustrate that C5 significantly reduces the tumor burden in two syngeneic murine tumor models with 4T1 and 67NR cells. These studies provide novel MPC inhibitors with potential for anticancer applications in monocarboxylate transporter 1–expressing breast cancer tumor models.

The onset of neoplasia results from numerous genetic mutations leading to uncontrolled growth, division, and proliferation. Hanahan and Weinberg (1) have defined several classical hallmarks of cancer, including evasion of apoptosis, self-sufficiency of growth signals, and insensitivity to antigrowth signals. Further, cancer cells adapt the ability to stimulate the growth of new blood vessels (angiogenesis) and evolve invasion and metastatic aptitude. In this regard, cancer cells exhibit limitless replicative potential forming genomically unstable cellular masses recognized as tumors (1). Within the tumor microenvironment, unequal distribution of oxygen and nutrients leads to diverse tissue types made up of highly heterogeneous cells where the metabolic and proliferative capability of the tumor depends largely on the extent of hypoxia (1, 2, 3, 4, 5). Oncogenic mutations that initiate the formation of the tumor mass lead to cells that are genomically unstable resulting in the replicative immortality and expansion of the malignancy (2, 6).

To expand on the classical hallmarks, numerous enabling characteristics and emerging hallmarks of cancer that support such replicative immortality within the diverse tumor microenvironment have been described (7). One of the important emerging hallmarks includes the ability of cancer cells to deregulate their energetics resulting in reprogrammed metabolism (5). To keep up with the large energy requirement of neoplastic proliferation, cancer cells exhibit marked metabolic shifts from what is observed in normal quiescent epithelial tissue (2, 5, 8, 7, 9). In nonmalignant cells, energy is obtained *via* an efficient mitochondrial oxidative phosphorylation (OxPhos), where 1 mol of glucose yields 36 mol of ATP. To do so, normal cells transfer high-energy electrons from NADH from several other biosynthetic pathways into the electron transport chain. During which, pyruvate (Pyr) enters the tricarboxylic acid (TCA) cycle through many coupled enzymatic redox reactions, which together reduces molecular oxygen to 2 mol of water. Warburg observed that cancer cells largely undergo a metabolic switch from OxPhos to the more energy-inefficient glycolysis, even under sufficient oxygen conditions (8, 10, 11, 12). To keep up with the high energy demand of cancer progression, glycolytic-related enzymes and transporters, including glucose transporters (GLUT1), hexokinase, glucose 6 phosphate dehydrogenase, monocarboxylate transporters (MCTs), and others, have been shown to be highly overexpressed in several malignancies (8). Specifically, lactate and Pyr flux through MCT1 (import) and MCT4 (export) have been associated with anabolic and catabolic cellular phenotypes, supporting cancer progression in certain cellular microenvironments (2, 6). This aerobic glycolysis, termed Warburg effect, has been observed in numerous cancer types under both oxygen-poor and -rich conditions and has emerged as a defining characteristic of cancer cell metabolism (8, 10, 11, 12).

Recent evidence in contrast to the Warburg effect postulates that cancer-associated stromal fibroblasts are stimulated to upregulate glycolysis, and the metabolic byproducts, namely lactate and Pyr, are shuttled from the stromal compartment to the cancer epithelial cells for mitochondrial OxPhos (11, 13, 14). This shift not only increases energy production but also enables metabolite cycling through the TCA cycle where metabolic intermediates are important for biomass generation (11, 13, 14). This phenomenon has been aptly termed the reverse Warburg effect and demonstrates the unique ability of tumors to recruit nonmalignant tissues and undergo metabolic symbiosis to obtain energy and biosynthetic starting materials in support of rapid and uncontrolled cell division.

One of the important molecular players in coupling glycolysis and mitochondrial OxPhos is the mitochondrial pyruvate carrier (MPC). The MPC facilitates the transport of cytosolic Pyr, either as a byproduct of glycolytic metabolism or influxed by MCTs, into the mitochondrial matrix (15, 16). The MPC1 and MPC2 genes encode two obligate protein subunits of the MPC that form a heterodimeric complex wherein both proteins are required for activity as loss of one leads to destabilization and degradation of the MPC complex (16). The MPC is found on the inner mitochondrial membrane and imports the metabolic end product of glycolysis, Pyr, into the mitochondrial matrix for incorporation into intermediary metabolism in the citric acid cycle (TCA) (15, 16). Thus, MPC couples the two major energetic pathways, glycolysis and OxPhos, for energetic and biosynthetic needs of the rapidly proliferating cancer cells. Importantly, recent evidence suggests that highly oxidative cancer cell types exhibit increased levels of mitochondrial respiration and anabolic processes that drive cancer cell proliferation (17). Thus, targeting of MPC has high therapeutic potential for the treatment of a wide variety of cancers that depend on increased mitochondrial metabolism. Breast cancer oncogenesis is associated with high levels of metabolic reprogramming and relies heavily on mitochondrial respiration and TCA cycle metabolism (2). Hence, we hypothesize in this study that inhibition of mitochondrial respiration through MPC inhibition will be a valuable treatment method to expand the scope of clinical breast cancer therapy.

We and others have recently developed new-generation *N,N-*dialkyl/diarylcyanocinnamic acid and aminocarboxycoumarin derivatives with potent inhibition properties of MCT-mediated lactate flux (18, 19, 20, 21). Recent evidence suggests that inhibition of mitochondrial Pyr flux leads to increased cytosolic Pyr concentrations that can modulate MCT-mediated lactate uptake (17). In this regard, we reason that our first-generation inhibitors may be acting on the MPC resulting in feedback-mediated inhibition of MCTs as we previously reported. In the current study, we have synthesized and evaluated a series of novel fluorinated aminocarboxycoumarin-based MPC inhibitors for the treatment of cancer.

## Results

### Design and synthesis of fluoro-aminocarboxycoumarin–based MPC inhibitors

We have reported on the synthesis and evaluation of novel *N*,*N*-dialkyl/diarylcyanocinnamic acid and aminocarboxycoumarin derivatives as inhibitors of MCT1 and MCT4 mediated lactate uptake for anticancer applications (18, 19, 20). From our previous studies, we have identified a lead candidate based on the *N,N*-diarylcyanocinnamic acid that potently inhibits lactate influx and exhibits significant tumor growth inhibition properties as a single agent in triple-negative breast cancer and colorectal tumor models (18, 20). However, we have found that the lead compound exhibits limited oral bioavailability and metabolic stability with low biological half-lives and rapid clearance (CL) rates. We attribute these pharmaceutical shortcomings to unsubstituted phenyl groups for rapid CYP450-mediated hydroxylation and subsequent phase-II metabolic elimination. Apart from us, Draoui *et al.* (21) also reported aminocarboxycoumarin derivatives as potent MCT1 inhibitors with significant single-agent activity in MCT1-expressing tumor models. Recently, Corbet *et al.* (17) demonstrated that the ability of these candidate compounds to inhibit lactate influx was due to MPC inhibition leading to intracellular Pyr levels that ultimately feedback regulate MCT-facilitated lactate flux. In this regard, we undertook a study on the structure–activity relationship of aminocarboxycoumarins to improve the drug-like properties of the lead *N*-methyl-*N*-benzyl aminocarboxycoumarin **C3** (Scheme S1*A*). Our previous experience (18) of low metabolic stability because of unsubstituted phenyl derivatives has prompted us to substitute fluorine atom in the para position of the benzyl group in the lead candidate. Fluorine or trifluromethyl (CF_3_) group substitution is routinely employed in medicinal chemistry to improve the stability of the metabolically vulnerable candidate compounds. We envisioned that fluorine or CF_3_ group substitution at the para position of the benzyl group of the previously identified lead candidate **C3** will lead to a novel candidate with better pharmaceutical properties. In this regard, we synthesized fluorinated aminocarboxycoumarins **C1**, **C2**, and **C4** (Scheme S1), which were then evaluated for their *in vitro* biological evaluation in breast cancer cell lines MDA-MB-231, 4T1, and 67NR.

### Drug candidates **C2–C4** inhibit Pyr-driven respiration in 4T1 cells

**Figure 1:**
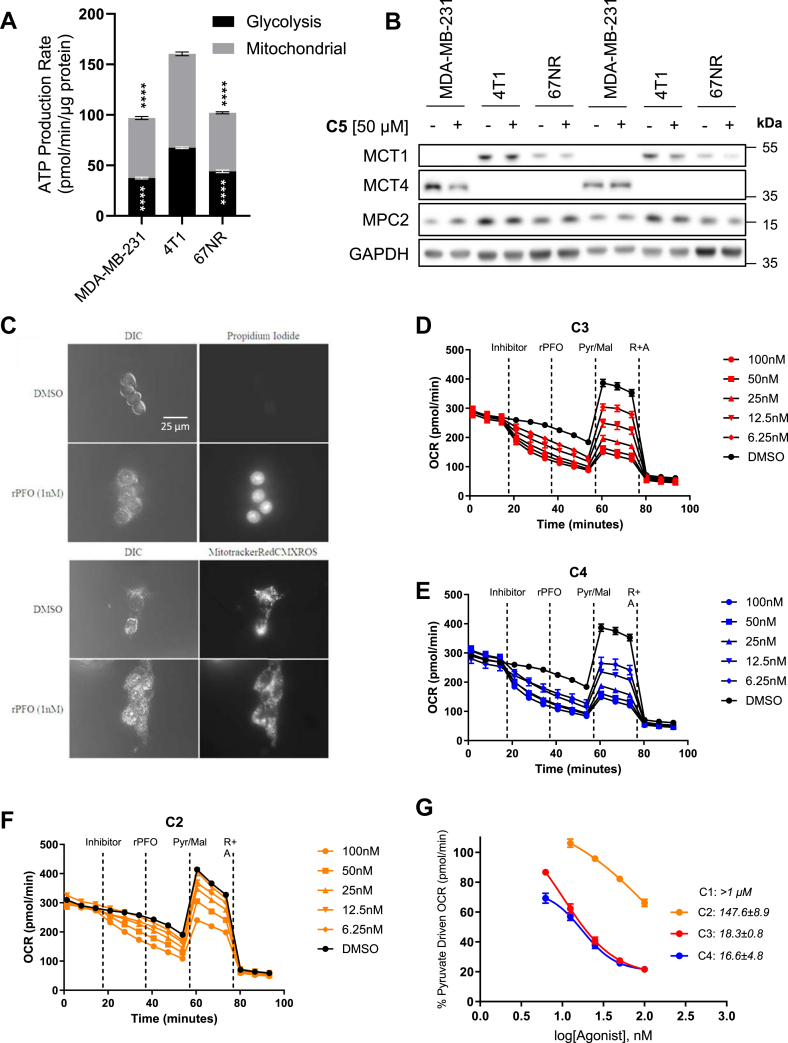
**Candidate aminocarboxy coumarin compounds inhibit pyruvate driven respiration in 4T1 cells.** Candidate compounds inhibit pyruvate (Pyr)-driven respiration in (*A*) highly oxygen-consuming and (*B*) MCT1- and MPC2-expressing 4T1 cells. Note MPC along with MCT1 and MCT4 expression status in MDA-MB-231, 4T1, and 67NR cell lines, which characterize differential cellular Pyr/lactate handling. MPC inhibition with candidate inhibitor **C5** does not alter MPC or MCT expression levels. Data presented represent two independent biological replicates, which are presented side by side in the Western blot (*B*). *C,* microscopy experiments illustrate that rPFO (1 nM) effectively permeabilized 4T1 cells as indicated by propidium iodide uptake without altering MitoTracker Red fluorescence. *D*–*F,* candidate compounds inhibit PPyr-driven respiration in permeabilized cells in a dose-dependent fashion enabling. *G,* dose–response curves to be generated and IC_50_ values to be calculated. All data are representative of the average ± SEM of at least three independent experiments. Statistics in Figure 1*A* are calculated by two-way ANOVA and are represented by ∗∗∗∗*p* < 0.0001, comparing 4T1 to both MDA-MB-231 and 67nr cell lines. MCT, monocarboxylate transporter; MPC, mitochondrial pyruvate carrier; rPFO, recombinant perfringolsyin O.

To validate that the candidate compounds retained the pharmacological properties of the parent aminocaroboxycoumarin **C3**, we evaluated their efficacy to inhibit Pyr-driven respiration in 4T1 breast cancer cell line. This cell line was found to exhibit substantially high rates of combined mitochondrial and glycolytic ATP production when compared with the isogeneic cell line 67NR and an established glycolytic breast cancer cell line, MDA-MB-231 (Fig. 1*A*). Further, MCT1 and 4 protein levels were assessed in these cell lines to characterize the Pyr/lactate importing or exporting phenotype, respectively (Fig. 1*B*). 4T1 and 67NR cell lines express MCT1 and are MCT4 null consistent with these cells preferentially importing and metabolizing Pyr and lactate (Fig. 1*B*). Conversely, MDA-MB-231 was found to be MCT1 null and MCT4 expressing indicating that this cell line exports Pyr/lactate, inconsistent with a mitochondrial Pyr phenotype (Fig. 1*B*). These results, combined with high bioenergetic rates and MPC2 expression levels (Fig. 1*B*), qualify 4T1 as a suitable cell line to investigate MPC inhibitors. To evaluate the ability of candidate compounds to inhibit Pyr-driven respiration because of MPC inhibition, we employed Seahorse XFe96-based respiratory experiments. In permeabilized cells, small polar metabolites, including Pyr, can be delivered directly to mitochondria independent of plasma membrane transport, and hence, the kinetics of mitochondrial Pyr-driven respiration can be measured directly. Recombinant perfringolsyin O (rPFO) is a cytolysin excreted by *Clostridium perfringins* that potently and acutely permeabilizes the plasma membrane of eukaryotic cells without impacting organelle membrane integrity. To test the ability of rPFO to permeabilize the plasma membrane in our model system, we employed epifluorescent microscopy experiments (Fig. 1*C*). Here, 4T1 cells were seeded in MatTek glass-bottom dishes and were incubated for 48 h for adherence. Cells were than exposed to rPFO (1 nM) and incubated for 30 min in growth media, a time point relevant to the exposure in Seahorse experiments. To validate membrane permeability, propidium iodide (PI) uptake was observed. Media were then aspirated and replaced with a mannitol/sucrose buffer (MAS buffer; 70 mM sucrose, 220 mM mannitol, 10 mM potassium phosphate monobasic, 5 mM magnesium chloride, 2 mM Hepes, and 1 mM EGTA) containing both PI and MitoTracker Red CMXROS (MTR)—a probe that binds and accumulates to the mitochondria as a function of membrane potential. PI enabled observation of membrane permeability as intact membranes do not allow PI uptake, and hence, MTR was utilized in these experiments to assess the effects of rPFO on mitochondrial viability as damaged mitochondria will exhibit a dim/diffuse fluorescence. These experiments revealed that rPFO potently (1 nM) and acutely (30 min) permeabilized the plasma membranes as indicated by PI uptake in rPFO-treated cells. Further, we observed that mitochondria remained viable with comparable MTR fluorescent intensity in rPFO cells when compared with the controls (Fig. 1*C*).

After validating the ability of rPFO to permeabilize 4T1 cells without damaging mitochondria, we sought to investigate the ability of candidate compounds to inhibit Pyr-driven respiration in these cells (Fig. 1, *D*–*G*). In this regard, 4T1 cells were seeded in Seahorse XFe96 well plates and were incubated to reach confluence (∼18–24 h) in growth media (+serum). Growth media were then aspirated, and cells were washed with MAS buffer to remove serum and endogenous metabolic substrates. Serum- and substrate-starved cells were then incubated for equilibration in MAS buffer in a non-CO_2_ incubator. Inhibitors, rPFO, and substrate milieus were prepared in MAS buffer to be injected in succession to allow for real-time observation of the effects of compound-treated cultures on oxygen consumption rates (OCRs) when compared with vehicle (dimethyl sulfoxide [DMSO])-treated cells. Equilibrated intact cells were allowed to establish basal respiratory rates, followed by the injection of candidate compounds **C1 to C4** at varying dose-titrated concentrations, respectively, and acute decreases in OCR were observed (Fig. 1, *D*–*G*). Compound exposure was performed in intact cells to avoid influence of rPFO on acute cellular targets. Cells were then exposed to rPFO, followed by the carbonyl cyanide-*p*-trifluoromethoxyphenylhydrazone (FCCP)–stimulated Pyr substrate milieu, which consisted of Pyr, malate (Mal), and dichloroacetate (DCA) to fuel uninhibited Pyr respiratory flux and maximal respiration. Mal and DCA were included to allow for continuous TCA cycle function without acetyl CoA feedback–mediated inhibition (Mal) or Pyr dehydrogenase–regulated inhibition (DCA) of Pyr uptake. Here, we observed a dose-dependent inhibition of Pyr-driven respiration in cells treated with the candidate compounds, allowing for logarithmic dose–response curves to be generated, and 50% inhibitory concentrations (IC_50_) to be calculated (Fig. 1, *D*–*G*). Finally, complex I and II inhibitors, rotenone and antimycin, A were injected to halt respiration and end the assay. These studies revealed that candidate inhibitors exhibited a range of IC_50_ values and gave important insights into structure–activity relationships. Interestingly, the bis-fluorophenyl **C1** completely lost activity when compared with parent **C3** as inhibitory concentration was not reached below 1 μM when compared with 18 nM of **C3** (Fig. 1*G*). Fluorosubstitution of **C4** did not alter the IC_50_ when of the parent with equipotent 16 nM inhibitory capacity (Fig. 1*G*). Interestingly, removal of the benzylic carbon in the *N-*methyl-*N-*fluorophenyl example **C2** resulted in decreased activity with an IC_50_ value of 147 nM, indicating a potentially important role of the *N*-methyl-*N*-benzyl template in **C3** and **C4**, respectively. In this regard, the fluoro-aminocarboxycoumarin derivative **C4** has been designated as the lead candidate for further investigation.

### Candidate compounds **C2, C3,** and **C4** specifically inhibits Pyr-driven respiration without substantial effects on other metabolic fuels

**Figure 2:**
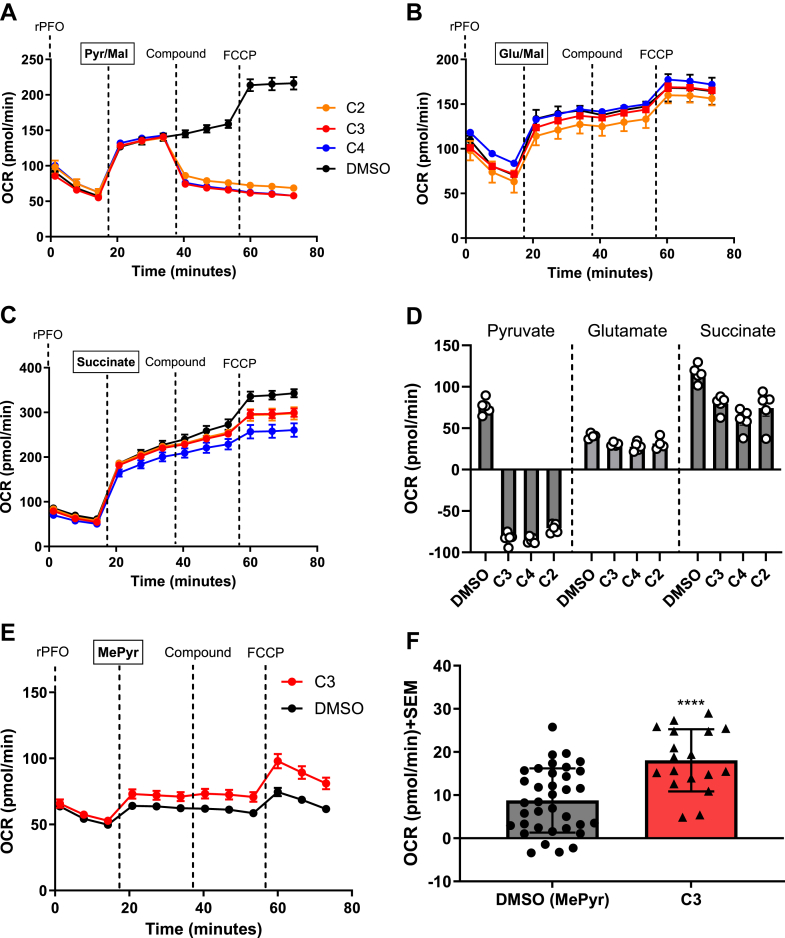
**Mitochondrial respiration effects of candidate compounds are specific to pyruvate transport.***A*–*D,* candidate compounds specifically inhibit pyruvate-driven respiration without substantial effects on glutamate- or succinate-driven respiratory processes (N = 1 experimental replicate, n = 5 technical replicates). *E* and *F,* methyl pyruvate reversed the inhibitory capacity of candidate compounds indicating acute MPC inhibition. All experiments are representative of at least three independent experiments (N = 3 and n = 16 technical replicates), and data represent the average ± SEM. One-way ANOVA was performed to indicate statistical significance between DMSO and compound-treated cultures (∗∗∗∗*p* < 0.0001). DMSO, dimethyl sulfoxide; MPC, mitochondrial pyruvate carrier.

Although our initial experiments and previous literature illustrate the ability of the candidate compounds to inhibit Pyr-driven respiration, we sought to investigate the ability of candidate compounds to inhibit respiration fueled by other metabolic substrates, glutamate and succinate (Fig. 2). Glutamate, similar to Pyr oxidation, results in NADH equivalents that fuel complex I-mediated respiration, and succinate metabolism offers FADH_2_ for electrons in complex II-driven respiratory processes. Hence, these metabolites play an important role in the overall viability of OxPhos. To further validate the mode of action of the candidate compounds, a series of similar Seahorse experiments were employed (Fig. 2, *A*–*C*). In these experiments, 4T1 cells were first permeabilized and were then offered metabolic substrates Pyr (Pyr/Mal; Fig. 2*A*), glutamate (Glu/Mal, Fig. 2*B*), or succinate (Fig. 2*C*), and the resulting OCR was observed. As described previously, Mal and DCA were included in the substrate milieu of glutamate respiration experiments. Cells were then offered candidate compounds (1 μM), and acute changes in OCR demonstrated that the synthesized compounds specifically inhibited Pyr-driven respiration without substantial effects on glutamate- or succinate-driven respiratory processes (Fig. 2, *A*–*D*). To validate that inhibition of Pyr respiration is due to mitochondrial Pyr uptake and not other Pyr-processing enzymes (*i.e.*, Pyr dehydrogenase), a parallel experiment using methyl pyruvate (MePyr) was employed (Fig. 2, *E* and *F*). MePyr is permeable to the mitochondrial membranes, where in the matrix, mitochondrial esterase hydrolysis activity provides Pyr. Hence, MePyr provides a means to deliver Pyr to the matrix independent of MPC activity, and reversal of the effects on Pyr-driven respiration in this regard demonstrates specific inhibition of MPC by candidate compounds. These experiments illustrated that Pyr-driven respiration effects of **C3** were completely reversed, indicating that **C4** and other similar fluoro-aminocarboxycoumarins **C2–C3** are acute and specific MPC inhibitors (Fig. 2, *E* and *F*).

### Michaelis–Menten kinetics and epifluorescent microscopy reveals insights into MPC binding, inhibitory capacity, and mitochondrial localization of candidate compounds

**Figure 3:**
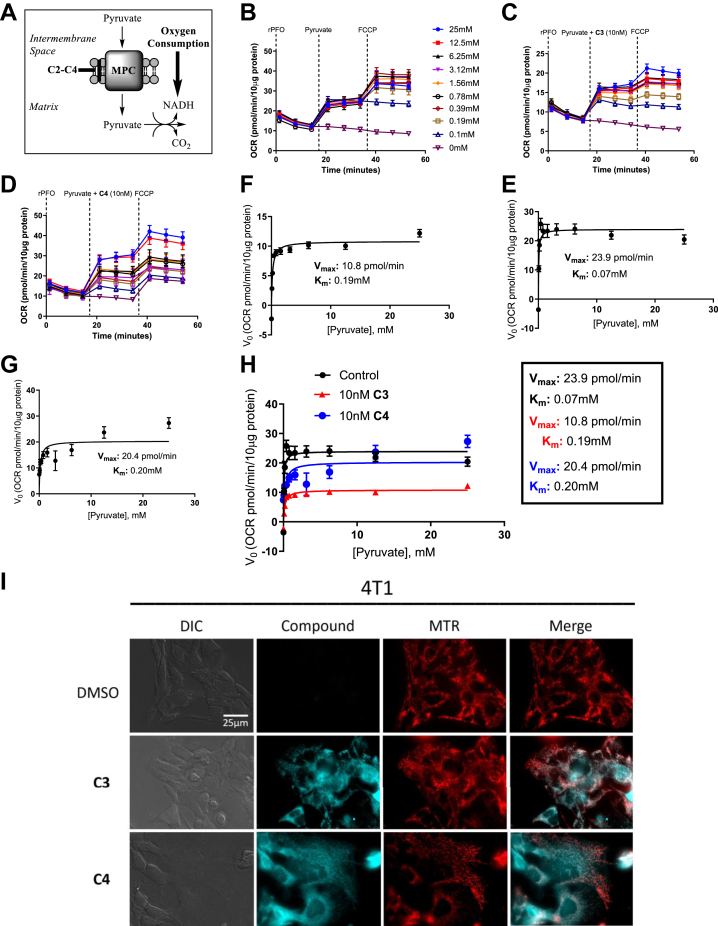
**MPC-binding characteristics of C3 are consistent with reversible covalent interactions with amino acids in the pyruvate-binding site.***A* and *B,* doubly activated olefins of cyanocinamic acid and carboxy coumarin template enable reversible covalent bonding with intracellular nucleophiles. *C*–*H,* Seahorse XFe96 assays illustrate Michaelis–Menten type kinetics of mitochondrial pyruvate uptake, with C3 demonstrating a “mixed” mode of inhibition based on decreased *V*_max_ and increased *K*_*m*_ when compared with control cultures. *A*–*H,* data represent the average ± SEM of N = 3 experimental replicates and n = 18 technical replicates. *I,* epifluorescent microscopy experiments reveal candidate compounds accumulate in the mitochondria in 4T1 cells. Compound fluorescence was captured under standard GFP filter sets where there was no observed fluorescence overlay into the *red* (MTR) channel. Images are representative of overall culture appearances (>5 fields of view) and of three independent experiments. All images were captured at the same magnification (see scale bar). All data are representative of the average ± SEM of at least three independent experiments. MPC, mitochondrial pyruvate carrier; MTR, MitoTracker Red CMXROS.

To further investigate the type of inhibition, we employed Michaelis–Menten experiments in modified Seahorse XFe96–based assays similar to described previously, and based on the model that Pyr uptake is directly coupled to NADH oxidation and oxygen consumption (Fig. 3*A*). In these experiments, 4T1 cells were permeabilized and were then administered varying concentrations of Pyr in the presence or the absence of 10 nM **C3** or **C4,** respectively (Fig. 3, *B*–*F*). A rise in OCR indicated Pyr-driven respiration, where subsequent addition of FCCP stimulated maximal respiration, and substrate-dependent increases in OCR were observed and normalized to protein content (Fig. 3, *B*–*F*). In this regard, Michaelis–Menten curves were generated using the FCCP-stimulated OCR in **C3-** or **C4**-treated and -untreated cells where Michaelis–Menten parameters of maximal velocity (*V*_max_) and half-maximal substrate concentrations (*K*_*m*_) were calculated (Fig. 3, *E*–*G*). Michaelis–Menten assumptions include direct and irreversible substrate and carrier interaction that results in the OCRs and is analogous to standard enzymatic Michaelis–Menten models (Fig. 3*A*). In the absence of inhibitor, 4T1 cells exhibited a *V*_max_ of 23.9 pmol/min and a *K*_*m*_ of 0.07 mM, whereas in **C3**-treated cells, *V*_max_ and *K*_*m*_ values were 10.8 pmol/min and 0.19 mM, respectively (Fig. 3, *E*, *F* and *H*). Thus, **C3** decreased the *V*_max_ and increased the *K*_*m*_, not consistent with competitive inhibition where high substrate concentrations titrate out the inhibitor to reach intrinsic *V*_max_ values. However, potential covalent bonding of inhibitor may limit the ability of Pyr to displace **C3** from the active site, resulting in the observed “mixed” mode of inhibition. Interestingly, **C4** increased the *K*_*m*_ of Pyr to 0.20 mM, similar to **C3**, but did not substantially alter *V*_max_, indicating a likely competitive mode of inhibition (Fig. 3, *G* and *H*).

To further investigate cellular localization of candidate compounds, epifluorescent microscopy experiments were employed (Fig. 3*I*). Conveniently, the aminocarboxycoumarin template offers fluorescent capabilities when excited in standard GFP excitation and emission settings, and hence, the cellular localization of **C3** and **C4** candidates can be observed. In this regard, 4T1 cells were seeded in MatTek glass-bottom dishes and were incubated for 48 h for adherence and flattening. Cells were then exposed to either **C3** or **C4** for 30 min, followed by MTR for 15 min at 37^o^C. Growth media were then aspirated and replaced with MAS buffer + 5% fetal bovine serum for imaging. Here, we observed that candidate compounds accumulated in granular structures, and significant overlay with MTR indicated substantial accumulation in the mitochondria consistent with MPC inhibition (Fig. 3*I*). Based on the lipophilic nature of C3 and C4, and the absence of mitochondrial targeting moieties (*e.g.*, triphenylphosponium), it is reasonable to predict that these compounds diffuse cellular and mitochondrial membranes and are retained in the mitochondria once bound to the MPC.

### Water-soluble and metabolically stable aminocarboxycoumarin MPC inhibitor **C5** exhibits enhanced water solubility and metabolic stability and elicits anticancer properties in murine breast cancer models

Encouraged by the retention of potent MPC inhibition properties of **C4**, we further replaced the F-atom in **C4** with CF_3_ group to synthesize **24** (Scheme S2*A*). CF_3_ group is extensively employed in drug discovery generation of lipophilic and metabolically more stable candidate compounds. In addition, one limitation of *N*,*N*-dialkylaminocoumarin derivatives is a lack of water solubility. To mitigate this issue, we sought to synthesize a salt of **24** with high solvating capacity (Scheme S2*B*). Starting with the carboxylic acid **24**, various salt forms were generated to increase the water solubility. A sodium salt was first generated but did not substantially increase the water solubility. Incorporating organic salt forms in drug development offers additional hydrogen bonding acceptor or donor groups and significantly enhances the solubility of organic compounds. Tris(hydroxymethyl)aminomethane (Tris base, **25**, Scheme S2*B*) was utilized because of its high solubility in water, low toxicity, and its p*K*_a_ is slightly above physiological pH (p*K*_a_ = 8.07), providing the ammonium counter ion at physiological pH. The candidate **C5** exhibited excellent water solubility properties of 10 mg/ml.

**Figure 4:**
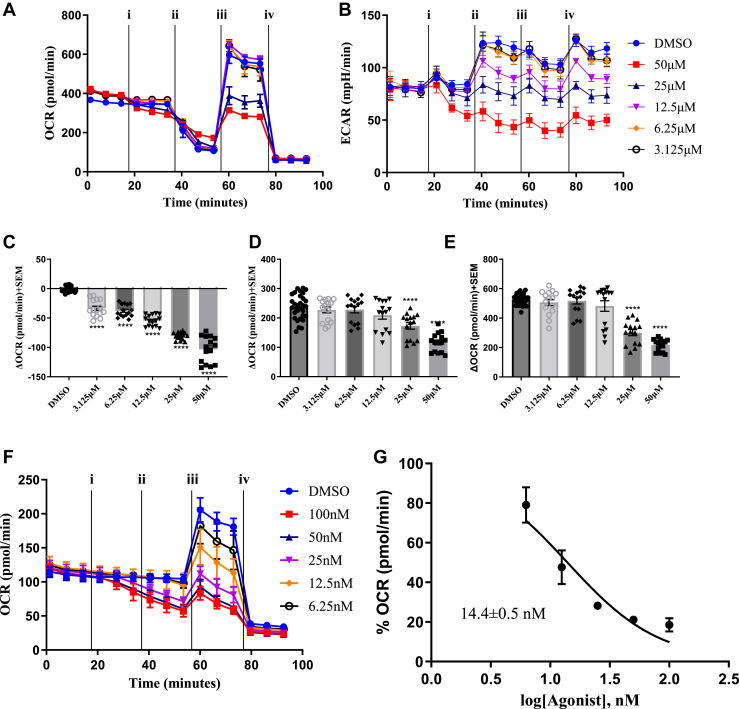
**Water-soluble and metabolically stable aminocarboxycoumarin MPC inhibitorC5 inhibits pyruvate driven respiration.** Seahorse-based mitochondrial stress test illustrates that C5 inhibits (*A*) mitochondrial respiration (oxygen consumption rates [OCRs]) and (*B*) compensatory glycolysis (extracellular acidification rates [ECARs]) in intact 4T1 cells. OCR and ECAR were measured following injection of (i) C5, (ii) oligomycin, (iii), FCCP, and (iv) a cocktail or rotenone and antimycin A. C5 reduces (*C*) acute OCR, (*D*) ATP production, and (*E*) maximal respiration. *F* and *G,* C5 inhibits pyruvate-driven respiration in permeabilized 4T1 cells. All data are representative of the average ± SEM of at least three independent experiments (N = 3, n = 15 technical replicates). One-way ANOVA was performed to indicate statistical significance between DMSO and compound-treated cultures (∗∗∗∗*p* < 0.0001). DMSO, dimethyl sulfoxide; FCCP, carbonyl cyanide-*p*-trifluoromethoxyphenylhydrazone.

To confirm that **C5** retained mitochondrial targeting capacity, we first carried out standard Seahorse mitochondrial stress tests, where assay media contain a variety of metabolic fuels, including glucose, Pyr, and glutamine (Fig. 4, *A*–*E*). Here, we observed that **C5** inhibited mitochondrial respiration in a dose-dependent fashion with notable decreases in oxygen consumption upon acute injection of compound (Fig. 4*C*), following oligomycin injection indicating decreases in ATP production (Fig. 4*D*), and following FCCP illustrating decreases in maximal respiration (Fig. 4*E*). Interestingly, we observed a simultaneous and dose-dependent decrease in compensatory glycolysis (extracellular acidification rates), suggesting a potential feedback inhibition of glycolysis in the MCT1-expressing 4T1 cells, consistent with MPC inhibition. Thus, we further characterized **C5** toward the inhibition of Pyr-driven respiration as described in Figures 1 and 2. Here, we found that **C5** inhibits Pyr-driven respiration to a similar extent when compared with both **C3** and **C4** with and IC_50_ value of 14.4 ± 0.5 nM in permeabilized 4T1 cells (Fig. 4, *F* and *G*). Consistent with **C3**, the **C5**-CF_3_ derivative did not alter mitochondrial respiration when offered glutamate or succinate (Fig. S2, *A*–*D*). Further, methyl Pyr was able to reverse the **C5-**inhibited Pyr-driven respiration, indicating that this inhibition is transport mediated (Fig. S2, *E*–*H*). Taken together, **C5** perturbs mitochondrial respiration consistent with MPC inhibition, qualifying this drug candidate for further anticancer efficacy studies.

### CRISPR–Cas9 experiments illustrate the necessity of MPC1 and MPC2 for C5 to inhibit Pyr-driven respiration

**Figure 5:**
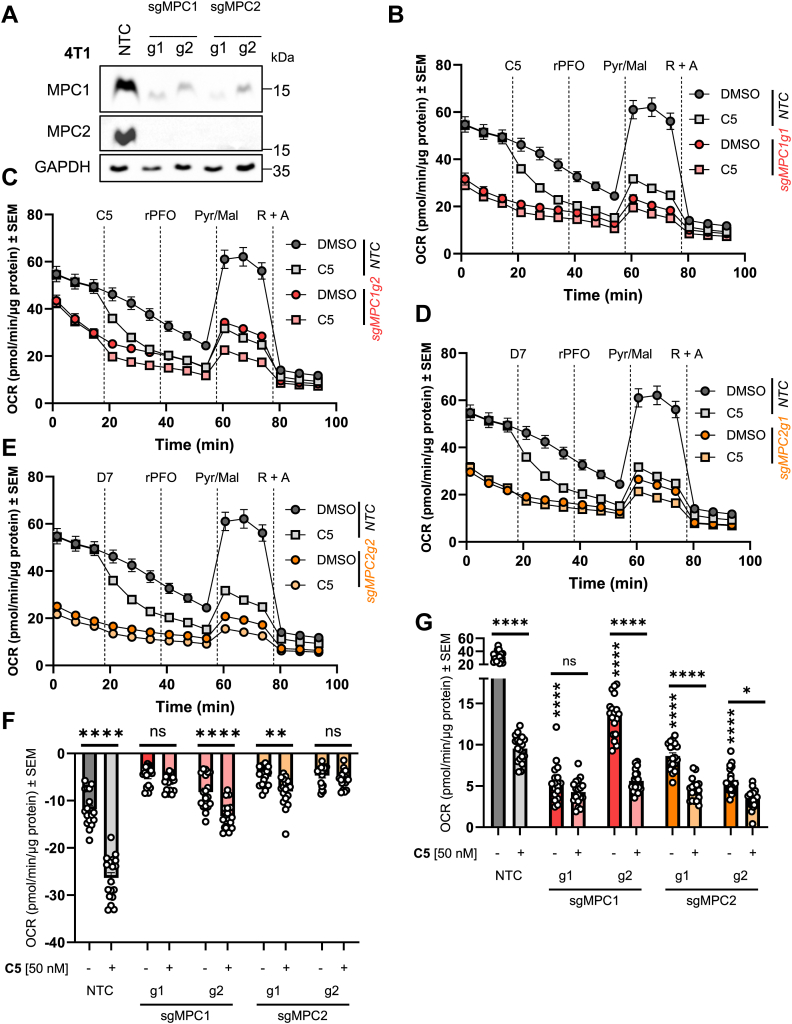
**MPC1 and MPC2 are necessary for C5 to inhibit pyruvate (Pyr)-driven respiration.***A,* Western blot of MPC1 and MPC2 indicating CRISPR–Cas9-mediated KO in 4T1 cells. g1, guide 1; g2, guide 2; NTC, nontarget control. Note targeted KO of MPC1 results in loss of MPC2 and *vice versa*. Images are representative of N = 1 biological replicate of each respective CRISPR–Cas9 cell line. Two guides (g1 and g2) targeting each MPC1 and MPC2 for controls. *B*–*E,* Pyr-driven respiration in permeabilized 4T1 cells with MPC WT (NTC) and MPC1/2 KO exhibit differential sensitivity to MPC inhibitor C5. Injection strategies similar to that presented in Figures 1 and 4. (*F*) acute response and (*G*) Pyr-driven maximal respiration of MPC1/2 WT and KO lines in the presence of vehicle (DMSO) and C5. Note attenuated responses to C5 in MPC KO lines. All data are representative of the average ± SEM of at least three independent experiments (N = 3, n = 15 technical replicates). One-way ANOVA was performed to indicate statistical significance between DMSO and compound-treated cultures (∗*p* < 0.05, ∗∗*p* < 0.01, and ∗∗∗∗*p* < 0.0001). DMSO, dimethyl sulfoxide; MPC, mitochondrial pyruvate carrier.

To further investigate the specificity of **C5** toward the MPC, we performed CRISPR–Cas9 KO of the MPC complex. The MPC is a heterodimer that consists of two obligate protein subunits, MPC1 and MPC2. Hence, we sought to knock-out both MPC1 and MPC2 to evaluate (1) the dependence of these proteins in facilitating Pyr-driven respiration in the mitochondria and (2) determine the necessity of these proteins for **C5** to inhibit this respiration. In this regard, we knocked-out MPC1 and MPC2 in 4T1 cells (Fig. 5*A*). Here, we found that KO of MPC1 led to loss of MPC2, and KO of MPC2 led to loss of MPC1, consistent with these proteins being obligate heterodimers. To test the necessity of these proteins to facilitate Pyr-driven oxidation, we performed similar respiration experiments as described in Figures 1 and 4 (Fig. 5, *B*–*E*). These experiments illustrated that loss of MPC1 (Fig. 5, *B* and *C*) and MPC2 (Fig. 5, *D* and *E*) resulted in substantial decreases in basal respiration rates and mimic in magnitude the respiration in control 4T1 cells injected with **C5**. In addition, we find that when compared with control cells, the acute response to **C5** injection is substantially attenuated in MPC1 and MPC2 KO cells (particularly MPC1 g1 and MPC2 g2 lines, Fig. 5*F*). Consistent with this, Pyr-driven maximal respiration (FCCP stimulated) is severely compromised in **C5**-treated control cells, but the effects of **C5** in the MPC-KO lines are largely diminished (Fig. 5*G*). Importantly, the maximal respiration stimulated by Pyr in MPC-KO lines is also minimal, and again mimics in magnitude that of **C5**-inhibited control 4T1 cells. These results strongly illustrate that the MPC complex is necessary for Pyr-driven respiration in 4T1 cells, and that the MPC is necessary for **C5** to inhibit Pyr-driven respiration, consistent with MPC being the molecular target.

### Human MPC molecular docking experiments indicate candidate inhibitors can bind to the MPC, which is associated with intracellular lactate accumulation and breast cancer cell death

To further investigate the inhibitory characteristics of candidate inhibitors, we carried out inhibitor docking studies to the recently determined cryo-EM structure of human MPC (22), both intermembrane space (IMS) and matrix-facing structures, to give structural insights into binding characteristics on MPC. Both the open-outward MPC and open-inward (shown) structures showed substantial water-accessible vestibules embedded in the central cavity of the protein (Fig. S1*A*). Ultimately, we focused on the open-inward matrix–facing cavity within the membrane-spanning helices of MPC consistent with the recently determined inhibitor-bound human MPC structures (22). This would be the location of the most favorable docking poses of each inhibitor. It should be noted that our inhibitors also have significant affinity for the outward-open (IMS facing) structure, but the docking poses were much more heterogeneous than poses determined for the inward matrix–facing structure.

For docking, **C2 to C5** structures were prepared in ChemDraw and Chem3D, with energy minimization (ChemOffice, version 19.1; PerkinElmer Informatics, 2020). Docking was carried out with inhibitors remaining flexible about rotatable bonds, whereas the MPC side chains were kept rigid. To avoid input bias, the initial docking search space included the entire human MPC structure except for the hydrophilic surface exposed to the inner membrane space, shown in Figure S1, *A* and *B*. The top docking pose of **C3** and **C5** is shown in Figure S1*A*, indicating the occupancy of the MPC open-inward pocket. The most favorable pose of inhibitor **C3** was estimated to bind to MPC with an affinity of −8.7 kcal/mol. In addition, lower energy poses occupied the same cavity with minor orientation differences. Further, in general, the lowest energy docking poses for each inhibitor displayed similar amino acid contacts in the binding pocket Fig. S1*C* (also depicted in Fig. S1, *D*–*E*).

Fig. S1*C* shows the amino acids within 4.5 A of inhibitors **C2, C3**, **C4,** and **C5** indicating some minor differences in pocket contacts. The amino acid side chains contacting C5, within 4.5 A, are shown in Figure S1*B*. Polar groups on one end of **C5**, carbonyl and carboxyl, are in position to make electrostatic interactions or hydrogen bonds to Asn33, Tyr62, and His84 from the MPC1 subunit and an electrostatic interaction or hydrogen bond with Lys49 and Asn100, respectively, with MPC2. Additional interactions include CH–pi interactions with Phe66 and Phe69 of MPC1 and a pi-stacking interaction with Trp82 of MPC2. Importantly, mutagenesis studies illustrate that Trp82 on MPC2 is necessary for UK5099 to bind and inhibit MPC (22). This interaction is highly conserved across C2, C3, C4, and C5, providing functional evidence that the candidate compound binding is inhibitory, consistent with UK5099. In addition, the more hydrophobic portion of the inhibitor makes extensive interactions with hydrophobic side chains, such as Leu, Ile, and Phe, in the binding pocket. The contacting amino acids are relatively consistent across the other inhibitors analyzed here, with minor differences. Across the four inhibitors docked to the matrix-facing MPC, the difference in estimated binding energy ranged from −7.9 to −9.4, most favorable for **C5**. In addition, there was slightly less heterogeneity in the top binding poses for **C5** in each of the MPC models. This may be the result of the introduction of additional steric restrains of the trifluorinated **C5**.

Docking of **C2** did show some obvious differences with respect to **C3**, **C4,** and **C5** raw docking results. **C2** poses comparable to those of **C3**, **C4,** and **C5** were consistently 0.8 to 1.5 kcal/mol less favorable. This difference equates to an approximately 10-fold difference in binding affinity, lower for **C2**. To add to this, **C2** shows more heterogeneity in its binding poses, including multiple binding poses outside the putative inhibitor-binding cavity, suggesting slightly less specificity and likely further lowering potency. The lower estimated binding energy and heterogeneity of docked poses are true despite **C2** interacting with similar amino acids in the binding pocket in the consensus binding pose. These observations are more than sufficient to explain the experimental loss of binding affinity of **C2**.

It is important to note that the consensus docking poses and amino acid contacts for all the inhibitors significantly overlapped with the position and contacts of the inhibitor UK5099 resolved in a matrix-facing human MPC cyro-EM structure (Protein Data Bank code: 9MNX). This binding mode, that is, open-inward, could also explain the mixed mode inhibitor kinetics observed here. In this scenario, inhibition is locking the MPC in a Pyr-incompetent binding mode. The inhibition kinetics might be further complicated by the fact that docking to open-outward, IMS-facing, MPC indicated significant energies of interaction with the Pyr-binding pocket. Although the binding modes did show significant heterogeneity from pose to pose, potentially suggesting lower specificity and lower affinity in the outward-open structure. It is also possible that the double-activated olefin present in the carboxy coumarin template offers potential nucleophilic addition capabilities with the variety of nucleophilic amino acid contacts identified. It is likely that this interaction is reversible because of the highly acidic alpha hydrogen with two adjacent electron-withdrawing groups, which is labile for elimination.

**Figure 6:**
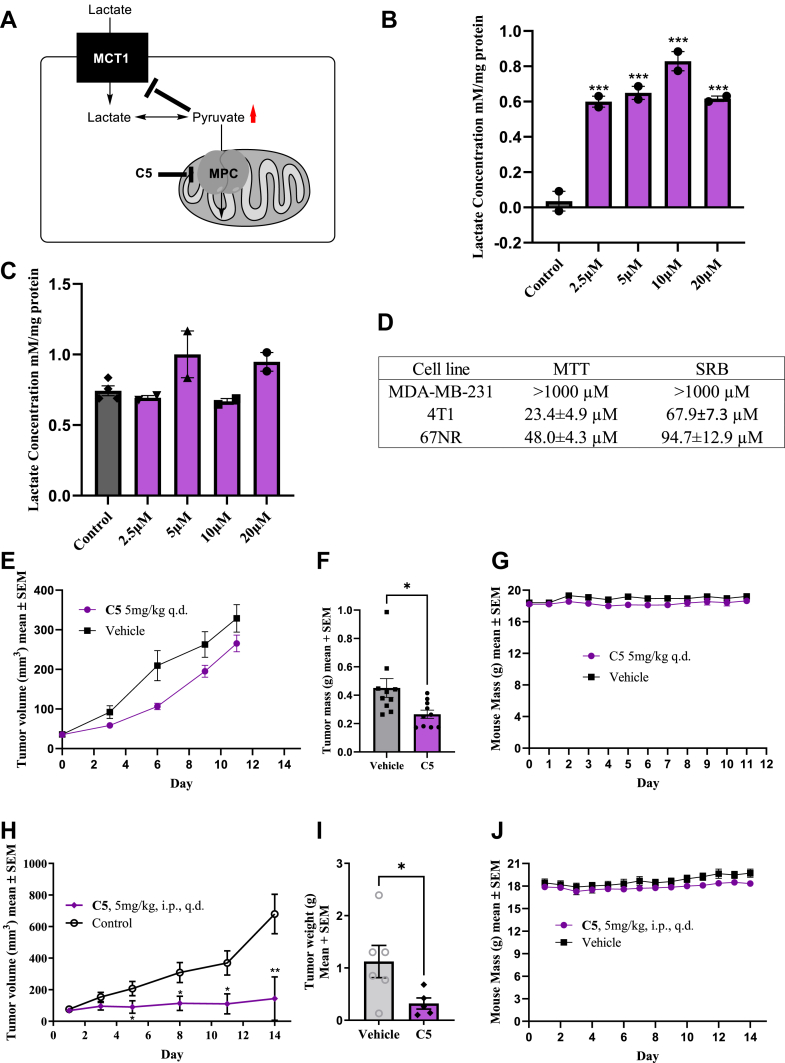
**C5 alters perturbs lactate flux and exhibits anticancer efficacy in *in vitro* models of breast cancer.***A,* model illustrating that inhibition of pyruvate uptake into mitochondria results in accumulation of intracellular lactate. C5 treatment results in the accumulation of lactate in (*B*) 4T1 but not (*C*) MDA-MB-231 cells. *D,* C5 is specifically toxic in 4T1 and 67NR when compared with MDA-MB-231, EC_50_ values represent the average ± SEM of at least three independent experiments. *E,* C5 inhibits 4T1 tumor volume and (*F*) tumor mass without affecting (*G*) mouse body weight. *H*–*J,* C5 exhibits anticancer efficacy in a 67NR tumor model. Data in (*B*) are representative of the average ± SEM of at least three independent experiments. One-way ANOVA was performed to indicate statistical significance between DMSO and compound-treated cultures (∗∗∗*p* < 0.001). Tumor volume and mass data, along with mouse weights (*E*–*J*), represent the average ± SEM of n = 10 mice. Statistical significance was calculated using the Mann–Whitney test (∗*p* < 0.05, ∗∗*p* < 0.01). DMSO, dimethyl sulfoxide.

As noted previously, **C5** treatment resulted in a dose-dependent decrease in glycolysis (Fig. 4*B*), which may be due to cytosolic accumulation of lactate upon MPC inhibition (Fig. 6*A*). To test this, we assayed intracellular lactate accumulation following **C5** treatment in MCT1-expressing 4T1 cells and MCT4-expressing MDA-MB-231 cells using commercially available lactate kits. MCT1 mediates lactate import, and MCT4 mediates lactate export, where 4T1 and MDA-MB-231 cells offer two cellular models of lactate accumulation. MCT4 expression is predicted to alleviate lactate accumulation upon MPC inhibition. Our results indicate that **C5** treatment results in a potent accumulation of lactate in 4T1 (Fig. 6*B*), but not MDA-MB-231 (Fig. 6*C*) cells, consistent with our hypothesis. It is possible that an intracellular accumulation of lactate can contribute to cell proliferation inhibition properties of **C5** in addition to its mitochondrial targeting capacity. Interestingly, consistent with lactate accumulation experiments, **C5** is selectively cytotoxic to 4T1 and isogeneic 67NR cell lines when compared with MDA-MB-231 (Fig. 6*D*), indicating that MPC inhibition may be particularly effective in MCT1-expressing cells. Based on the potent MPC and enhanced cell proliferation inhibition properties, the water-soluble derivative **C5** was designated as lead candidate for *in vivo* studies.

### Lead candidate compound **C5** exhibits suitable pharmacokinetic properties, is well tolerated in healthy mice, and exhibits tumor growth inhibition properties in mouse models of breast cancer

To examine the potential of candidate compound **C5** to be translated to *in vivo* anticancer efficacy studies, we first sought to determine the pharmacokinetic (PK) properties in BALB/c mice. PK properties of parameters of **C5** in BALB/c mice were evaluated using three mice per time point, plasma samples were collected after intravenous (IV) treatment of 1 mg/kg and PO treatment of 5 mg/kg of **C5** in both male and female mice (Fig. S3*A*). Using these data, PK parameters such as half-life (*t*_1/2_), *T*_max_, *C*_max_, apparent volume distribution at steady state (*V*_ss_), CL, and bioavailability use Pheonix WinNolin 7.0 in a noncompartmental analysis method. IV dosing allows for analysis under the assumption of total systemic absorption (Fig. S3*A*). Under this assumption, it was found that **C5** has a *t*_1/2_ of 3.15 and 3.65 h with CL of 26.61 ml/min/kg and 34.30 ml/min/kg in female and male mice, respectively. **C5** was found to have *V*_ss_ of 6.92 l/kg in female mice and 10.24 l/kg in male mice. Volumes of distribution above 5 l/kg are considered medically to be high values, indicating that **C5** has a high *V*_ss_ in both male and female mice and may have a likelihood to leave plasma and distribute into tissues. However, with a noncompartmental analysis method, the tissue-specific accumulation of **C5** is not known at this time. In oral dosing of **C5**, the *t*_1/2_ was found to be 4.41 and 4.04 h in female and male mice, respectively. In addition, the bioavailability was found to be 124.7% and 123.8% in female and male mice, respectively (Fig. S3*A*). It is not possible to obtain a bioavailability over 100%; however, we can extrapolate these values to mean that **C5** has close to 100% bioavailability.

To test if the addition of trifluoromethyl in **C5** led to improved metabolic stability when compared with the parent compound **C3**, we evaluated the intrinsic CL rates of **C3** and **C5** in human liver microsomes (Table S1). Here, compounds were incubated in the presence of isolated human liver microsomes, and the presence of **C3** and **C5** was determined by HPLC–MS/MS at a variety of time points. Half life and intrinsic CL rates (Cl_int_) were determined and establish relative stability when compared with controls with high and low metabolic stability, respectively (Table S1). These results indicate high levels of metabolic stability of both **C3** and **C5** with half-life's exceeding the experimental endpoints (>120 min), consistent with metabolically stable controls imipramine and propranolol (Table S1). However, **C5** exhibits a ∼two-fold increase in Cl_int_ when compared with that of **C3**, which indicates that **C5** requires twofold larger volume of buffer to be cleared. This result suggests an increased metabolic stability of **C5** when compared with **C3**, which may be attributed to trifluoromethyl protection from liver oxidation.

After obtaining PK parameters of **C5**, we carried out a general tolerability dose escalation study *via* intraperitoneal injection to determine a maximum tolerated dose for future tumor efficacy studies (Fig. S3*B*). As we did not carry out pharmacodynamic studies on **C5**, we wanted to administer compound through intraperitoneal injection to ensure a large systemic absorption of the drug. In this experiment, body weight changes of >10% from the start of the study represented a drug burden and was considered a toxic dose. In addition, mice were monitored daily for any changes in behavior and grooming to determine if there were any effects seen outside body weight loss (BWL). In CD-1 mice, dosing began at 5 mg/kg for 7 days. After 7 days, dosing was increased to 6 mg/kg; however, at this dose, there were acute respiratory effects and lethargy observed in the mice deeming it a toxic dose. Therefore, because of no observed behavioral changes and normal body weights, 5 mg/kg was considered the maximum tolerated dose *via* intraperitoneal injection (Fig. S3*B*).

Due to **C5** being 100% bioavailable, we also carried out maximum tolerated dose studies in healthy severe combined immunodeficient mice with oral dosing. Severe combined immunodeficient mice were administered **C5** at 10, 20, 40 mg/kg daily in groups of five mice for 21 days (Medicilon; study #03092-22002, Fig. S3*C*). In this experiment, body weight changes of >10% from the start of the study represented a drug burden and was considered a toxic dose. In addition, mice were monitored daily for any changes in behavior and grooming to determine if there were any effects seen outside the BWL. It was found that there were not any significant changes in body weight or behavior in all the treatment groups; therefore, 40 mg/kg was considered the maximum tolerated dose for oral administration (Fig. S3*C*). A 7-day single-dose acute toxicity study was carried out after oral administration of **C5** at 10, 25, and 40 mg/kg. During the 7 days, there were no abnormal changes in behavior, food consumption, and no signs of clinical toxicity. The lower tolerability of **C5** when administered intraperitoneally (Fig. S3*B*) when compared with oral dosing may suggest a higher systemic tissue absorption of **C5**. Although oral PKs indicated 100% bioavailability (*i.e.*, in blood, Fig. S3*A*), the amount of drug to be absorbed in tissue likely did not reach the level absorbed *via* i.p injection, rendering i.p. a less-tolerated dosing strategy. After the study ended, no gross pathology, including general cardiotoxicity, was observed after organ resection (Medicilon; study #03092-22002).

To further evaluate potential cardiotoxic effects, we evaluated the ability of **C5** to inhibit the human Ether-a-go-go (hERG) potassium channels commonly associated with off-target cardiotoxicity of small-molecule therapeutics. Here, using ion channel electrophysiology, we evaluated the ability of **C5** to inhibit hERG channel function when compared with known hERG channel inhibitor verapamil. These results illustrated that **C5** did not inhibit hERG channel function at concentrations that it inhibits the MPC (Fig. S4, *A* and *B*). Taken together, normal cardiac histology and lack of hERG channel inhibition properties provide encouraging evidence that **C5** does not cause appreciable cardiotoxicity.

To further evaluate the anticancer efficacy of **C5**, we performed *in vivo* syngraft models using two isogeneic murine breast cancer cell lines 4T1 and 67NR derived from distinct sites from the same mouse. 4T1 cell line is characterized as highly metabolically plastic and aggressive, whereas 67NR is less-so. Gratifyingly, MPC inhibition characteristics were conserved in the 67NR cell line (Fig. S3*D*). For this experiment, 0.1 ml of 4T1 cells were injected into the flanks of BALB/c mice with a 1:1 mixture of Matrigel and PBS containing 12,500 cells/mouse. After tumors reached 50 mm^3^, mice were randomly placed into groups. **C5** was then administered as a 10% DMSO/water solution at 5 mg/kg daily for 11 days. After 11 days, vehicle control mice began to ulcerate, so mice were sacrificed, and tumors were resected. After 11 days of treatment, it was found that **C5**-treated mice saw a ∼20% reduction in tumor volume (TV) compared with vehicle-treated control (Fig. 6*E*). However, after tumor resection, it was found that **C5**-treated groups caused a ∼41% reduction by mass when compared with vehicle-treated control (Fig. 6*F*). Mice weights remained constant throughout the duration of the study (Fig. 6*G*). For the 67NR model, 500,000 67NR cells in a 1:1 solution of Matrigel and PBS for a total volume of 0.1 ml were administered into the flank of BALB/c mice. Tumors were allowed to grow until they were ∼100 mm^3^ and randomly grouped. At that time, **C5** was administered 5 mg/kg intraperitoneally once daily for 14 days. After 14 days, there was a ∼79% reduction in TV in **C5** groups as compared with vehicle treated (Fig. 6*H*). At the end of the study, tumors were resected, and tumor mass was recorded. Compared with vehicle control, **C5** was found to reduce tumor mass by ∼71% (Fig. 6*I*). Mouse body weights remained stable during the 14-day treatment period (Fig. 6*J*).

## Discussion

The MPC is in its relative infancy in terms of tractability for clinical translation, particularly in cancer. Based on its important role in intermediary mitochondrial metabolism, it has been placed as an important regulator of metabolic disease phenotypes, including cancer, where there is a vast literature supporting reprogrammed energetic and biosynthetic pathways. In this regard, the utility of MPC inhibitors in cancer has largely been context dependent, and so far, rather underexplored, with the current study the first to illustrate single-agent activity in a breast cancer syngraft model. MPC inhibition has been found to have radiosensitizing effects in models of cervical cancer (17). Here, and consistent with our findings (Fig. 6), MPC inhibition led to toxic accumulation of intracellular lactate/Pyr and OXPHOS inhibition. This is consistent with our finding that MPC inhibitors exhibit toxicity in MCT1-expressing tumors, where intracellular lactate accumulation is favored. *In vivo*, MPC inhibition as a single agent offered moderate anticancer efficacy but durable synergy when combined with radiation therapy (17). The limited single-agent activity in this case could be explained by high levels of metabolic plasticity that is exhibited by tumors, where MPC inhibition has been shown to enhance compensatory branched chain amino acid metabolism (23) and others (24). In our models, we compare two isogeneic cell lines, 4T1 and 67nr, which differ in their levels of metastatic capacity, differentiation state, and metabolic plasticity. Specifically, 4T1 cells are more metabolically plastic than 67nr cells (24), which may explain the enhanced efficacy of MPC inhibition with **C5** observed in 67nr when compared with 4T1 syngraft models (Fig. 6), as a single agent.

MPC inhibition also provides efficacy in a context-dependent manner in gliomas (25). Here, disruption of TCA cycle flux caused decreases in cellular acetyl CoA levels, which associated with decreases in global histone acetylation. In this particular context, glioma progression depends on dedifferentiation events that are associated with epigenetic changes and histone marks (25). MPC inhibition decreased the ability of cells to alter glioma cell differentiation state in a histone acetylation–dependent manner; illustrating the important role of the MPC in not only bioenergetic and synthetic processes but also epigenetics. In this regard, it is quite possible that the enhanced efficacy of **C5** in 67nr may be a function of epigenetics that is more potent in the more differentiated and nonmetastatic state, than the dedifferentiated and metastatic 4T1 isogeneic line.

Taken together, the MPC plays important roles in regulating energetics, biosynthesis, and epigenetics, and its utility as an anticancer agent is context dependent. The current study illustrates that MPC inhibition has promise as a single agent in treating certain subtypes of breast cancer and can be rationally combined with other chemo- and radiation therapies to realize its full potential.

## Conclusions

In conclusion, we have synthesized and evaluated a novel series of fluoro-substituted aminocarboxycoumarin derivatives with potent MPC inhibition properties in low nanomolar concentrations. Seahorse-based respiratory experiments illustrated that candidate compounds specifically inhibit Pyr-driven respiration without substantially affecting other metabolic fuels consistent with MPC inhibition. Further, aminocarboxycoumarin-binding characteristics were found to be consistent with reversible covalent bonds with amino acids in the Pyr-binding domain. Epifluorescent microscopy experiments illustrated that candidate compounds predominantly accumulate in the mitochondria. We have further carried out maximum tolerated dose, single-dose acute toxicity, and PK studies in Balb/C mice with the lead candidate **C5**. Anticancer efficacy studies in two murine syngeneic triple-negative breast cancer models with 4T1 and 67NR cells showed significant tumor growth inhibition. These studies illustrate that **C5** exhibits tumor growth inhibition properties in murine breast cancer models and exhibits favorable pharmaceutical properties including water solubility and metabolic stability to be considered as a suitable anticancer agent for further preclinical development.

The authors acknowledge limitations and bias with the current study. The molecules synthesized in this study have been biased by our previous work in that the parent compound, C3, has been previously shown to inhibit lactate flux in cancer cells. In addition, the study is biased in that we assume the MPC to be the only molecular target. Although we illustrate that MPC is necessary for inhibitor activity, we do not rule out other off-target effects of compound treatment in its anticancer mechanism. Beyond the scope of this current study, future directions may include genome-wide or metabolically targeted CRISPR screens, which may expand the scope of target engagement of **C5** and related drug candidates. In addition, proteomic experiments, such as proteome integral solubility alteration, would approach target engagement in an unbiased and full-proteome fashion. However, the current study provides valuable and empirical data on the inhibition mechanisms and anticancer efficacy of this class of aminocarboxycoumarin compounds.

## Experimental procedures

### Synthesis of fluoro-aminocarboxycoumarin derivatives and C5

Synthetic procedures and compound characterization are included in the supporting information.

### Cell lines and culture conditions

4T1, 67nr, and MDA-MB-231 cells were obtained from American Type Culture Collection. Cells were maintained using standard culture conditions at 37 °C and 5% CO_2_.

### Seahorse XFe96–based respiratory experiments

Permeabilized cell assays were performed using rPFO as described previously. 4T1 cells were seeded (20,000 cells/well) onto Seahorse XFe96 well plates and incubated overnight in growth media at 37 °C and 5% CO_2_ for adherence. On the day of the assay, growth media were aspirated and replaced with MAS buffer (70 mM sucrose, 220 mM mannitol, 10 mM potassium phosphate monobasic, 5 mM magnesium chloride, 2 mM Hepes, and 1 mM EGTA) after 3X rinse of growth media to remove serum and endogenous metabolic substrates, and incubated at 37 °C in a non-CO_2_ incubator. Respective inhibitor and substrate milieu's were prepared in MAS buffer for port injections A–D at 8X, 9X, 10X, and 11X the target cell concentrations to account for intrinsic dilution factor of *in situ* injections of each port. For some experiments (Fig. 4, *A* and *I*), compound **2** was injected in port A, followed by rPFO (1 nM) in port B, followed by respective substrate cocktails (FCCP stimulated) in port C and rotenone and antimycin A (0.5 μM) in port D. In other experiments (Fig. 4, *C*–*H*), permeabilization was initiated prior to port A injection during the MAS buffer wash phase, followed by substrates, test compound, and FCCP (0.125 μM). Final substrate concentrations for specific tests were as follows: (5 mM Pyr , 0.5 mM Mal, 2 mM DCA; 10 mM glutamate, 0.5 mM Mal, 2 mM DCA; 10 mM succinate, 2 μM rotenone; 20 mM methyl Pyr, 5 mM Pyr, 0.5 mM Mal, and 2 mM DCA). ATP rate assays and mitochondrial stress tests were performed according to the manufacturer's (Agilent) instructions.

### Western blotting

MDA-MB-231, 4T1, and 67NR cells were seeded in 35 mm dishes (5 × 10^5^ cells/dish) and were incubated for 24 h, and whole cell lysates were collected in radioimmunoprecipitation buffer. After denaturation at 95 °C for 10 min in radioimmunoprecipitation buffer, protein concentration was determined and equilibrated using the bicinchoninic acid assay (ThermoScientific), and 20 μg of protein was separated by electrophoresis in 4 to 12% polyacrylamide gels. Proteins were electrophoretically transferred to polyvinylidene fluoride membranes for 90 min at 100 V and stained with Ponceau to verify transfer quality and equal protein loading. After blocking the membranes with 10% w/v nonfat milk in Tris-buffered saline with Tween-20 (TBST) for 1 h at 35°C, membranes were incubated overnight at 4 °C with primary antibodies (MCT1; Santa Cruz, catalog no.: sc-365501, 1:1000 dilution; MCT4, Santa Cruz, catalog no.: sc-376140, 1:1000 dilution; MPC1, Cell Signaling, 1:1000 dilution, catalog no.: 14462; MPC2, Cell Signaling, 1:1000 dilution, catalog no.: 46141; GAPDH, Cell Signaling, 14C10, 1:1000 dilution; TBST, 0.5% nonfat milk protein). Membranes were rinsed three times with TBST and incubated with the respective secondary goat antimouse IgG (1:10,000 dilution) or goat anti-rabbit IgG (1:10,000 dilution) horseradish peroxidase–conjugated antibodies (Cell Signaling). Membranes were again washed three times with TBST and exposed to SuperSignal 60 West luminol enhancer and stable peroxide solution (ThermoScientific) for 2 min. Chemiluminescent images were obtained using a LicorFC imager. Images shown are representative of two separate experiments obtained from independent biological samples.

### CRISPR–Cas9 experiments

CRISPR–Cas9 experiments were performed using the GeCKO protocol. Single guide RNA (sgRNA) guides were cloned into lentiCRISPR-V2 plasmids containing the puromycin resistance vector (Addgene; catalog no.: 98290). sgRNA-cloned lentiCRISPR-V2 plasmids were then transformed into Stbl3-competent *Escherichia coli* cells and were purified. To generate virudes, human embryonic kidney 293T cells were seeded in 6-well dishes (6 × 10^5^ cells/well) and reverse transfected using PolyFect (Qiagen; catalog no.: 301105). Transfection was carried out using 880 ng of lentiCRISPR-V2 with respective target- or nontargeting control sgRNA, 600 ng psPAX2 (Addgene; catalog no.: 12260), and 300 ng pMD2 (Addgene; catalog no.: 12259). Following 24 h transfection, media were replaced with Dulbecco's modified Eagle's medium, and virus was generated for an additional 48 h. Lentiviral medium was then harvested, filtered (0.45 μm), and added with polybrene (10 μM) onto 4T1 cells (3 × 10^5^ cells/well, adhered overnight). Eighteen hours after transduction, cells were reseeded into 10 cm dishes, and puromycin (5 μg/ml) was added. Cells were selected for 5 to 7 days before used for experiments. The following sgRNA sequences were used: sgMPC1: sg1 5′-ACTTCCGGGACTATCTCATG-3′; sg2 5′-GCAAAGCGGCGGACTATGTC-3′; sgMPC2: sg1 5′-GGCCACCTACCACCGACTCA-3′, and sg2 5′-CCTGCCGGGTGGTTGTAAAG-3′.

### Molecular docking to human MPC1/2 complex

Recent cryo-EM structures of human MPC1/2 (22) were used for computational docking studies, docking compounds C3, C4, C2, and C5 to both inward-open matrix-facing MCP and outward-open inner membrane space facing MPC. AutoDock Vina (Vina) was used to dock the MPC inhibitors to structures (26, 27). The inhibitor docking search area was set intentionally broad, encompassing nearly the entire MPC structures and extending several angstroms toward the inward or outward-open aqueous surface. Due to the large search space, the Vina exhaustiveness parameter was increased to 100, the number of binding modes to return was set to 20, and the maximal energy differential was set to 6. Other variables such as adding hydrogens to the protein and removing nonpolar hydrogens from the inhibitors were set to default values in Vina and initially configured in Chimera. Docked inhibitor poses were viewed with UCSF Chimera, and the top consensus poses for compounds C3, C4, C2, and C5 were selected for further analysis. The number of poses nearly identical to the most favorable docked pose generated, estimated by position, orientation, and MPC residues contacted for each inhibitor and MPC model, was used as a surrogate for binding specificity. Final analyses and comparisons were summarized based on the open-inward matrix–facing MPC structure as high-resolution structures suggest that is the most probable inhibitor-binding mode.

### Epifluorescent microscopy

4T1 cells (5 × 10^4^ cells/ml) were seeded in glass-bottom dishes (MatTek Corp; part no.: P35G010C) and incubated for 48 h. C3 or FAAC2 were then added (10 μM), and cells were again incubated for 6 h 30 min and MTR (Invitrogen; M7512, 100 nM) was added 15 min prior to microscopic fluorescent imaging. Media were then aspirated and replaced with MAS + 5% fetal bovine serum for imaging. Cells were then examined and photographed using a Nikon TE2000 epifluorescent microscope and camera. The images shown are representative of at least five fields of view of three separate experiments and captured under the same 60× oil emersion differential interference contrast lens, see scale bar.

### L-lactate accumulation assay

MDA-MB-231 or 4T1 cells were seeded on a 6-well plate at 150,000 cells/well in their respective culture media and incubated for 3 days (37 °C, 5% CO_2_). On the third day, media were aspirated, and wells were rinsed twice with cold PBS. After rinsing, phenol red–free RPMI1640 (10% fetal bovine serum and penicillin–streptomycin [50 μg/ml]) was added with five treatment concentrations and one control and incubated for 24 h (37 °C, 5% CO_2_). After 24 h, extracellular media were removed, and cells were washed three times with ice-cold PBS. At this time, 1 ml of PBS was added to each well and underwent freeze–thaw lysis at −80 °C. Media in each well were aliquoted and centrifuged (16,000*g*, 10 min, 4 °C). The lysate supernatant was then aliquoted, and pellet was discarded. l-lactate concentration was then determined using Eton Biosciences L-Lactate assay kit 1 by mixing lysate sample with L-lactate assay solution in duplicate, incubating for 30 min (37 °C, 0% CO_2_) and measuring absorbance of enzyme produced INT formazan at 490 nm using a BioTek Synergy 2 plate reader. Absorbance values were compared with an L-lactate standard curve concomitantly ran with test samples to determine lactate concentration in each sample. Lactate concentrations were normalized to total protein using a Pierce Bicinchoninic Acid Protein Assay Kit.

### 3-(4,5-Dimethylthiazol-2-yl)-2,5-diphenyltetrazolium bromide cell proliferation inhibition assay

Confluent cell cultures were treated with trypsin and resuspended at 5 × 10^4^ cells/ml. To a 96-well plate, 100 μl of the 5 × 10^4^ cells/ml solution were added and allowed to incubate at 37 °C, 5% CO_2_ for 24 h. Compounds were then added and allowed to incubate for 3 days. At this time, 10 μl of 3-(4,5-dimethylthiazol-2-yl)-2,5-diphenyltetrazolium bromide (5 mg/ml) was added to the 96-well plate and further incubated for 4 h. Following the 4-h incubation with 3-(4,5-dimethylthiazol-2-yl)-2,5-diphenyltetrazolium bromide, 100 μl of SDS (0.1 g/ml, 0.01 N HCl) was added, and 96-well plates were allowed to incubate for an additional 4 h. Absorbance values were then taken at 570 nm using a Biotek Synergy 2 plate reader. Treatment absorbances are taken as percentages of the average untreated absorbances and plotted against log(concentration) in GraphPad Prism 10 (GraphPad Software, Inc) to generate effective concentration values where 50% of the cells are not proliferating (EC_50_).

### Sulforhodamine B cell proliferation inhibition assay

Cells were seeded (3 × 10^4^ cells/well) in 48-well plates and incubated overnight (37 °C, 5% CO_2_). Test compounds were then exposed to cells in duplicate in serial dilution fashion and were incubated for an additional 72 h (37 °C, 5% CO_2_). Media were then aspirated, and cells were washed three times with cold PBS and left overnight to dry fix. About 100 μl Sulforhodamine B (0.5% w/v in 1% aqueous acetic acid) was added to each well and incubated at 37 °C for 1 h. Sulforhodamine B was rinsed with 1% acetic acid solution and dried. Dyed cells were then lysed with Tris (10 mM, pH 10), and absorbance readings were recorded at 540 nm of each well. Treatment absorbances are taken as percentages of the average untreated absorbances and plotted against log(concentration) in GraphPad Prism 10 to generate effective concentration values where 50% of the cells are not proliferating (EC_50_).

### Tumor efficacy study in a 67NR syngraft

Female BALB/c (Charles River Laboratories) were administered 67NR at 500,000 cells/mouse into their flank as a 1:1 solution of Matrigel and PBS for a total volume of 0.1 ml. Tumors were grown until they reached a volume of ∼100 mm^3^ and were then randomized into groups (n = 6). Mice were administered intraperitoneally either vehicle control (10% DMSO, 90% sterile water) or C5 at 5 mg/kg once daily for 14 days. Body weights were recorded daily and monitored for behavior and grooming patterns. A BWL > 10% indicated a halting of treatment until weight recovery. If a BWL > 20% was observed, that mouse would be euthanized. TV (mm^3^) was calculated as follows: TV = (a × b^2^)/2, a as the tumor length and b as the tumor width. The tumors were measured every 2 to 3 days using a caliper in two dimensions. Mice were euthanized at the end of the 14-day treatment period where tumors were then resected and weighed. The study was carried out under Institutional Animal Care and Use Committee protocol 2106-39167A. Statistical analysis was carried out *via* unpaired *t* test using GraphPad Prism 10 (∗*p* < 0.05, ∗∗*p* < 0.01, ∗∗∗*p* < 0.001, and ∗∗∗∗*p* < 0.001).

### Tumor efficacy study in a 4T1 syngraft

Female BALB/c (Charles River Laboratories) were administered 67NR at 12,500 cells/mouse into their flank as a 1:1 solution of Matrigel and PBS for a total volume of 0.1 ml. Tumors were grown until they reached a volume of ∼50 mm^3^ and were then randomized into groups (n = 6). Mice were administered intraperitoneally with either vehicle control (10% DMSO, 90% sterile water) or C5 at 5 mg/kg once daily for 11 days. Body weights were recorded daily and monitored for behavior and grooming patterns. A BWL >10% indicated a halting of treatment until weight recovery. If a BWL > 20% was observed, that mouse would be euthanized. TV (mm^3^) was calculated as follows: TV = (a × b^2^)/2, a as the tumor length and b as the tumor width. The tumors were measured every 2 to 3 days using a caliper in two dimensions. Mice were euthanized at the end of the 14-day treatment period where tumors were then resected and weighed. The study was carried out under Institutional Animal Care and Use Committee protocol 2106-39167A. Statistical analysis was carried out *via* Dunnett's one-way ANOVA using GraphPad Prism 10 (∗*p* < 0.05, ∗∗*p* < 0.01, ∗∗∗*p* < 0.001, and ∗∗∗∗*p* < 0.001).

### PK assessment of C5 in a noncompartmental analysis model using BALB/c mice

In a contract organization Medicilon, 35 male and 35 female BALB/c mice were randomized and put into two groups (n = 15) per sex for each route of administration (4 total groups). Extra mice were used for collection of blank plasma. Ten designated time points were utilized of 0.083, 0.25, 0.5, 1, 2, 4, 6, 8, 10, and 24 h. C5 (10% DMSO, 90% sterile water) was administered IV at 1 mg/kg (5 ml/kg) *via* tail vein injection (n = 3 per arm) and orally *via* gavage after overnight fasting at 5 mg/kg (10 ml/kg) (n = 3 per arm). After administration of C5, 100 μl of blood was taken from the submandibular or other suitable vein at the designated time points, cardiac puncture was used for terminal bleeding. Each time point utilized three mice per group, and each mouse had two blood draws. The blood samples were collected in K_2_EDTA tubes on ice, centrifuged at 6800*g* (6 min, 4 °C) to generate plasma samples. The plasma samples were stored below −70 °C until LC–MS/MS analysis. The LC–MS/MS method development and sample analysis were performed by Medicilon testing facility, and the accuracy of >66.7% of the quality control samples should be within 80 to 120% of their nominal values. After data acquisition, noncompartmental analysis parameters were calculated by Pheonix WinNonlin 7.0.

### Intrinsic CL in liver microsomes

The liver microsome intrinsic CL studies were contracted to Eurofins labs, and the procedure was carried out as previously described (28). Metabolic stability, expressed as percent of the parent compound remaining, was calculated by comparing the peak area of the compound at the time point relative to that at time 0. The half-life (T1/2) was estimated from the slope of the initial linear range of the logarithmic curve of compound remaining (%) *versus* time, assuming the first-order kinetics. The apparent intrinsic CL (CLint, in μl/min/mg) was calculated according to the following formula: CLint = 0.693/T1/2∗(mg protein/μl or million cells/μl or pmol CYP isozyme/μl).

### General tolerability of C5 in CD-1 mice administered intraperitoneally

Female CD-1 mice (Charles River Laboratories) were randomly assigned into groups (n = 6 mice per group), and mice were administered with C5 intraperitoneally at 5 mg/kg or vehicle control (10% DMSO, 90% sterile water) once daily for 7 days. Body weights were recorded daily and monitored for behavior and grooming patterns. A BWL > 10% indicated a halting of treatment until weight recovery. If a BWL > 20% was observed, that mouse would be euthanized. Dosing was increased to 6 mg/kg on day 8, but significant acute respiratory effects were observed indicating toxicity. Due to this acute effect, the study was terminated, and all mice were euthanized. The study was carried out following Institutional Animal Care and Use Committee protocol 2211-40546A.

### General tolerability of C5 in BALB/c mice administered orally

Female BALB/c mice (Beijing Vital River Laboratory Animal Technology Co, Ltd) were randomly assigned into groups (n = 5 mice per group). Four groups were made, and mice were administered vehicle control (10% DMSO, 90% sterile water) or C5 orally at 10, 20, and 40 mg/kg once daily for 21 days. Body weights were recorded daily and monitored for behavior and grooming patterns. A BWL > 10% indicated a halting of treatment until weight recovery. If a BWL > 20% was observed, that mouse would be euthanized. The study was carried out by Medicilon Preclinical Research (Shanghai) LLC (study #CNL2203P) using license no.: SCXK (Beijing) 2021-0006 and animal certificate no.: 110011220106449863.

### Single oral dose acute toxicity

24 Balb/c mice (12 males and 12 females, SPF grade) purchased from Zhejiang Vital River Laboratory Animal Technology Co, Ltd were weighed and grouped to three mice per group. Each mouse was administered a single oral dose of vehicle (10% DMSO/water), 10 mg/kg C5, 25 mg/kg C5, or 40 mg/kg. Mice were clinically examined 0.5 to 1 h postdose and every day for 7 days. Clinical examination observations included evaluation of the skin, fur, eyes, ears, nose, oral cavity, thorax, abdomen, external genitalia, limbs and feet, respiratory and circulatory effects, autonomic effects such as salivation, and nervous system effects, including tremors, convulsions, reactivity to handling, and bizarre behavior. Mouse mass and food consumption were recorded daily with average food consumption per animal = the sum of food consumption per cage/the total number of surviving animals in this cage. After 7 days, mice were euthanized, and necropsies were carried out to observe any gross pathologies. During the necropsy, mice were observed for any external abnormalities, subcutaneous masses, or abnormalities in the abdominal, pelvic, thoracic, and cranial cavities. Additionally, organs were resected and observed for abnormalities. This study was carried out by Medicilon Preclinical Research (Shanghai) LLC (study #03092-22002) using license no.: SCXK (Zhe) 2019-0001 and animal certificate no.: 20220927Abzz0619000899 and 20220927Abzz0619000338.

### hERG inhibition assay

The hERG inhibition was contracted to Eurofins labs, and the procedure is briefly as follows: The parameters measured were the maximum tail current evoked on stepping to 40 mV and ramping back to −80 mV from the test pulse. All data were filtered for seal quality, seal drop, and current amplitude. The peak current amplitude was calculated before and after compound addition, and the amount of block was assessed by dividing the test compound current amplitude by the control current amplitude. Control data are the mean hERG current amplitude collected 15 s at the end of the control period; test compound data are the mean hERG current amplitude collected 15 s at the end of each 5-min test compound application for each concentration. IC_50_ values of C5 and verapamil were calculated comparing the current obtained following incubation of test compound at each respective dose compared with the vehicle (DMSO) control. After whole cell configuration is achieved, the cell is held at −80 mV. The cell is depolarized to +40 mV for 500 ms and then to −80 mV over a 100 ms ramp to elicit the hERG tail current. This paradigm is delivered once every 5 s to monitor the current amplitude. All compounds were tested in the presence of 0.1% nonionic surfactant and at approximately room temperature.

### Statistical analysis

Experimental replicates (N) were obtained from independent biological samples and are represented as averages of (n) technical replicates. Statistical analysis between two samples was done using Student's *t* test or one- and/or two-way ANOVA with multiple comparisons where appropriate. Mann–Whitney test was utilized for the *in vivo* efficacy studies where normal distribution was not assumed. Statistical significance was determined with *p* value < 0.05∗, < 0.01∗∗, < 0.001∗∗∗, and < 0.0001∗∗∗∗ as noted within the respective figure legends.

### Ethical considerations

The general tolerability study in CD-1 was approved and in compliance with the University of Minnesota's Institutional Animal Care and Use Committee (2211-40546A, April 13, 2024). This study was performed in accordance with the relevant guidelines and regulations, and all protocols were approved by the University of Minnesota. The BALB/c general tolerability study with oral dosing was carried out by a contract organization Medicilon Incorporated. Animals were purchased from Beijing Vital River Laboratory Animal Technology Co, Ltd. The study was carried out using license no.: SCXK (Beijing) 2021-0006 and animal certificate no.: 110011220106449863. The BALB/c single oral dose acute toxicity study, which was carried out by a contract organization Medicilon Incorporated. Animals were purchased from Zhejiang Vital River Laboratory Animal Technology Co, Ltd. The study was carried out by Medicilon Preclinical Research (Shanghai) LLC (study #03092-22002) using license no.: SCXK (Zhe) 2019-0001 and animal certificate no.: 20220927Abzz0619000899 and 20220927Abzz0619000338. The 67NR and 4T1 tumor syngrafts were approved and in compliance with the University of Minnesota's Institutional Animal Care and Use Committee (2106-39167A, October 11, 2024). This study was performed in accordance with the relevant guidelines and regulations, and all protocols were approved by the University of Minnesota.

## Data availability

All data generated or analyzed during this study are included in this published article (and its supporting information files).

## Supporting information

This article contains supporting information (27).

## Conflict of interest

The authors declare that they have no conflicts of interest with the contents of this article.

## Supporting information

Supporting information

Uncropped Blots
