# Supplementary material for: Development of novel mitochondrial pyruvate carrier inhibitors for breast cancer treatment
Source: J Biol Chem. 2025 Jul 16;301(8):110486. doi: 10.1016/j.jbc.2025.110486 (PMC12355554; doi:10.1016/j.jbc.2025.110486)

**SUPPLEMENTARY INFORMATION**

**Scheme S1.** Synthesis of **C1**-**C4**. (**A**) Chemical structures of candidate MPC inhibitors **C3**, and C1, C2, and C4. Arrows on **C3** denote points of synthetic modification. (**B**) To synthesize the corresponding fluoro-aminocoumarin derivatives, the hydroxyl group of starting material 3-aminophenol **1** was first protected with ^t^butyldimethylsilyl chloride (TBS-Cl) to obtain the corresponding silyl protected amino phenol **2**. At this stage, **2** was utilized in the synthetic scheme of **C1** and **C2** with varying synthetic protocols. Towards the synthesis of **C1**, **2** was subjected to amination onto *p*-fluoro-bromobenzene under catalytic Pd(OAc)_2_/P(^t^Bu)_3_ conditions in the presence of KO^t^Bu (**Scheme 1B**). The resulting bis-fluorophenyl-o-silyl protected **3** was further deprotected under acidic conditions to provide the corresponding 3-(bis(fluorophenyl)amino)phenol **4**. Direct Vilsmeier-Haack formylation of **4** resulted in the corresponding 4-(bis(4-fluorophenyl)amino)-2-hydroxybenzaldehyde **5**. Compound **5** was condensed with diethyl malonate under Knoevenagel conditions in the presence of piperidine and acetic acid in methanol. Subsequent hydrolysis using sodium hydroxide resulted in the product **C1**. The synthesis of **C2** was initiated with selective mono-methylation of **2** with methyl iodide to obtain 3-((tert-butyldimethylsilyl)oxy)-N-methylaniline **6**. Amination of **6** onto *p*-fluoro-bromobenzene under catalytic Pd(OAc)_2_/P(^t^Bu)_3_ conditions in the presence of KO^t^Bu was again performed resulting in the 3-((tert-butyldimethylsilyl)oxy)-N-(4-fluorophenyl)-N-methylaniline **7** (**Scheme 1B**). Acid-mediated deprotection of **7**, followed by Vilsmeier-Haack formylation of **8** provided the respective 4-((4-fluorophenyl)(methyl)amino)-2-hydroxybenzaldehyde **9**. In parallel fashion, Knoevenagel condensation of **9** with diethyl malonate in the presence of piperidine and acetic acid yielded the candidate compound **C2**. (**C**) A slightly modified scheme was utilized to obtain **C3** and **C4**. Initial silyl protection of 3-aminophenol **1** was performed using ^t^butyldiphenylsilyl chloride (TBDPS-Cl) to provide the corresponding o-protected aminophenol **10.** Reductive amination of **10** with either benzaldehyde or p-fluorobenzaldehyde resulted in the corresponding benzyl or fluoro-benzyl amines **11a** and **11b**, respectively. Methylation utilizing methyl iodide under basic conditions provided the equivalent *N*-methyl **12**, which was further deprotected under acidic conditions providing the 3-(benzyl(methyl)amino)phenol **13**. Above mentioned Vilsmeier-Haack formylation conditions provided the corresponding aldehyde **14**. Knoevenagel condensation of **14** with diethyl malonate, followed by hydrolysis provided the **C3** and **C4**, respectively.

**
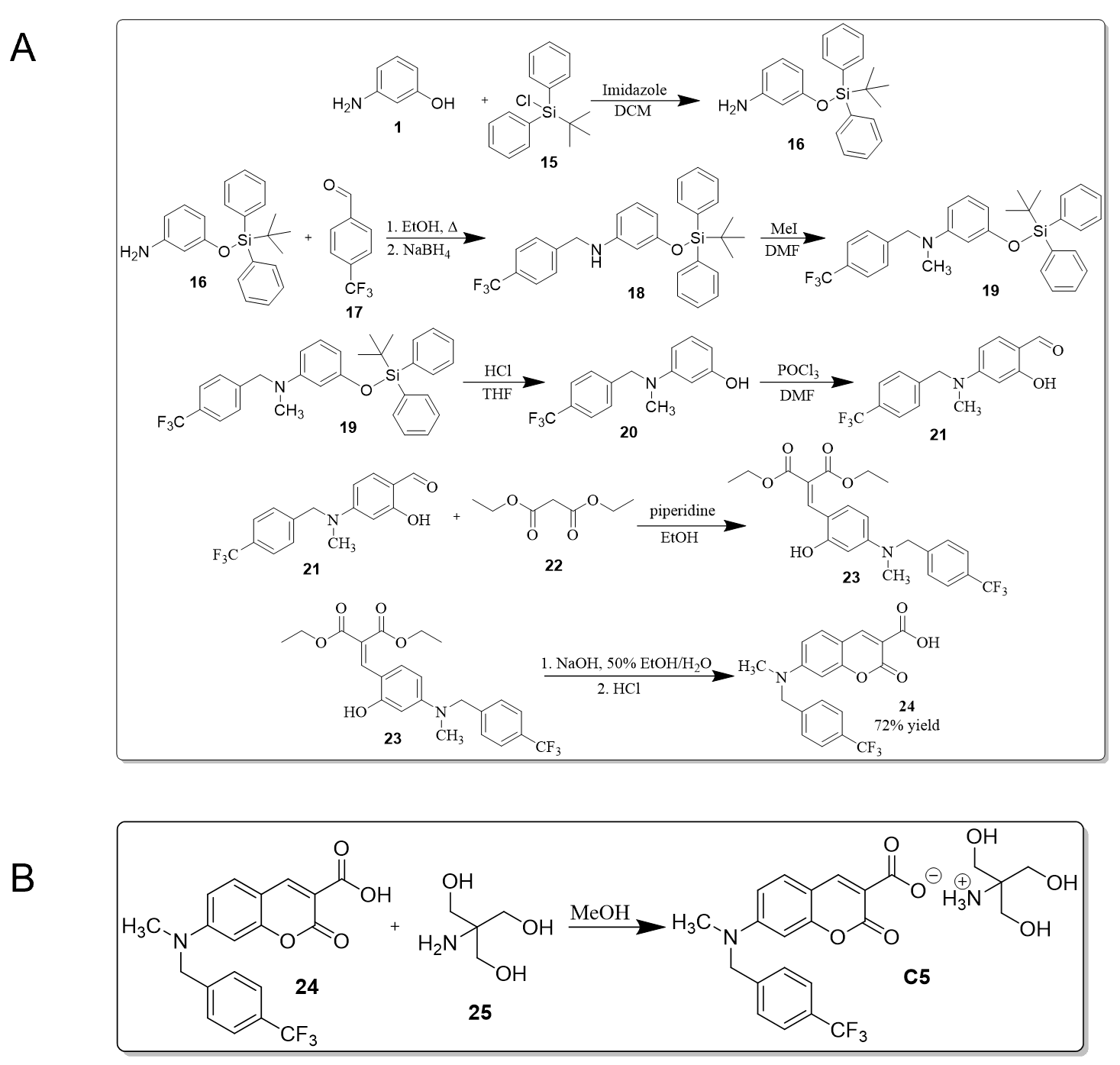
**

**Scheme S2.** Synthesis of trifluoromethyl substituted **24** and its corresponding tris-salt **C5**. (**A**) The synthesis of **24** began with silylation of 3-aminophenol **1** with ^t^butyldiphenylsilyl chloride **15** in the presence of imidazole base in DCM (**Scheme 2A**). This newly protected amine **16** was then subjected to reductive amination by sodium borohydride after condensation with 4-(trifluoromethyl)benzaldehyde **17** in refluxing ethanol. The aryl mono substituted silyl protected amine **18** was then methylated using methyl iodide in DMF to yield **19**. This disubstituted amine **19** was then deprotected in THF using HCl. The newly formed phenol **20** was subjected to Vilsmeier-Haack formylation using POCl_3_ in DMF. This salicylaldehyde **21** then underwent Knoevenagel condensation with diethyl malonate **22** in the presence of piperidine in ethanol. The condensation product **23** then underwent NaOH hydrolysis followed by acidification and cyclization using HCl to afford **24**, in ~72% yield. (**B**) To synthesize the water soluble **C5**, the carboxylic acid **24** was dissolved in methanol followed by the addition of tris base **25**. After stirring for 1 hour a bright yellow solid precipitated out, filtered, and rinsed with cold MeOH to yield the tris base form, **C5**.

**
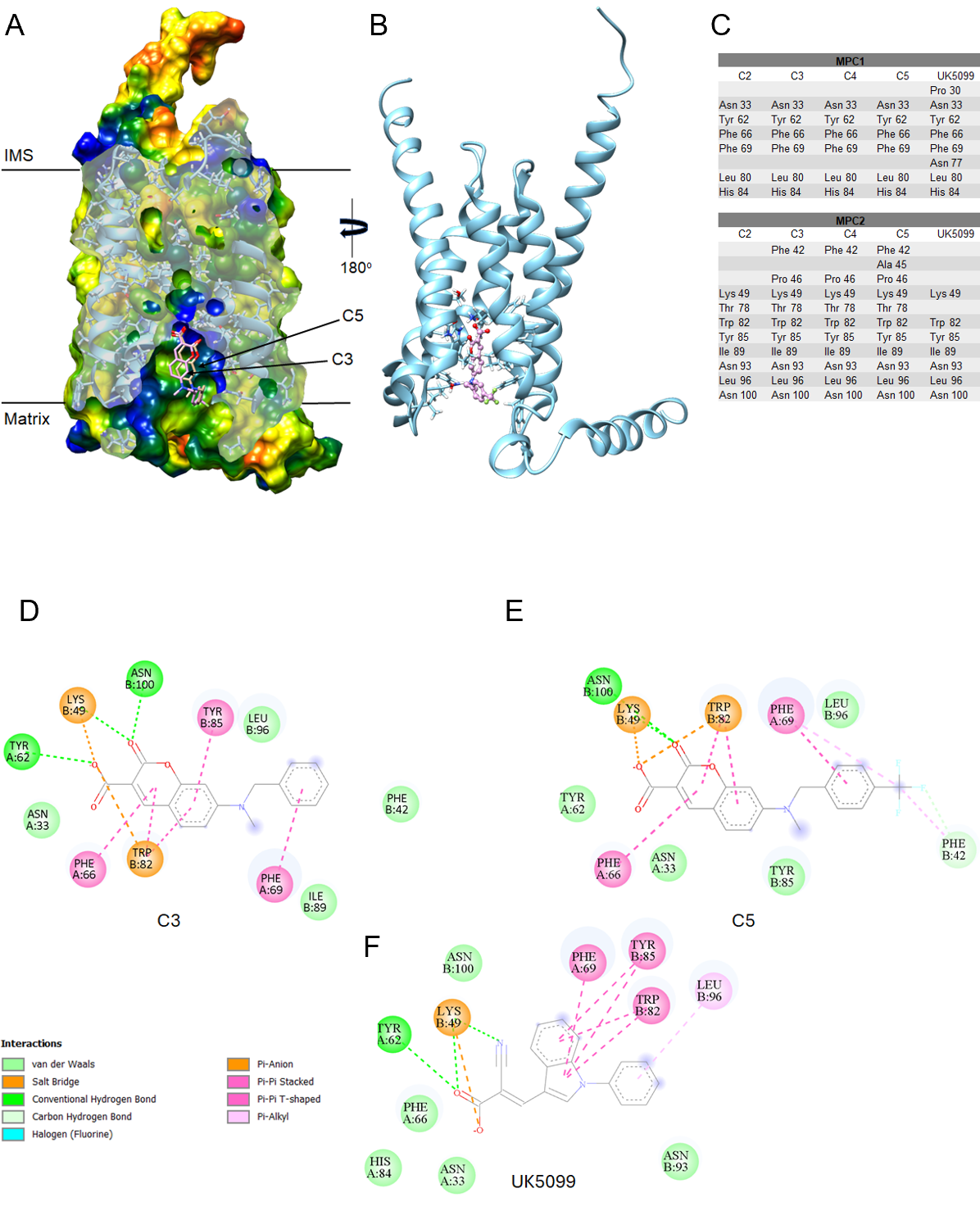
**

**Figure S1.** Computational modeling and inhibitor docking studies of MPC with C3 reveal interactions with amino acids in the pyruvate binding site. (**A**) Electrostatic surface of Human MPC homology model with docked inhibitor C3. The structure has been clipped to better show the binding cavity for inhibitor C3 and approximate positioning of the inner-mitochondrial membrane is indicated. Left: Lowest energy docked consensus pose for 7ACC. Center: Top-down view (intermembrane space facing) of docked C3. Similar outcomes were observed for C4 and C2. Right: Contacts between inhibitors C3, C4 and C2 and amino acids within 4.5A in their docked poses. The part of the model residing in the mitochondrial matrix was not included in the docking search space. (**B**) Consensus binding mode of inhibitor C5. Left: Electrostatic surface of MPC with lowest energy pose of docked C5. Center: Ribbon diagram of Human MPC showing amino acids within 4.5A in contact with inhibitor C5, ball and stick representation. (**C**) Summary of amino acids within 4.5A of inhibitors C5 and C3. Inhibitor docking studies illustrating the two-dimensional depiction of the lowest energy consensus binding poses of inhibitor (**D**) C3, (**E**) C5 and known MPC inhibitor (**F**) UK5099. Types of interactions are indicated in the legend. Figure made with Discovery Studio Visualizer [28].


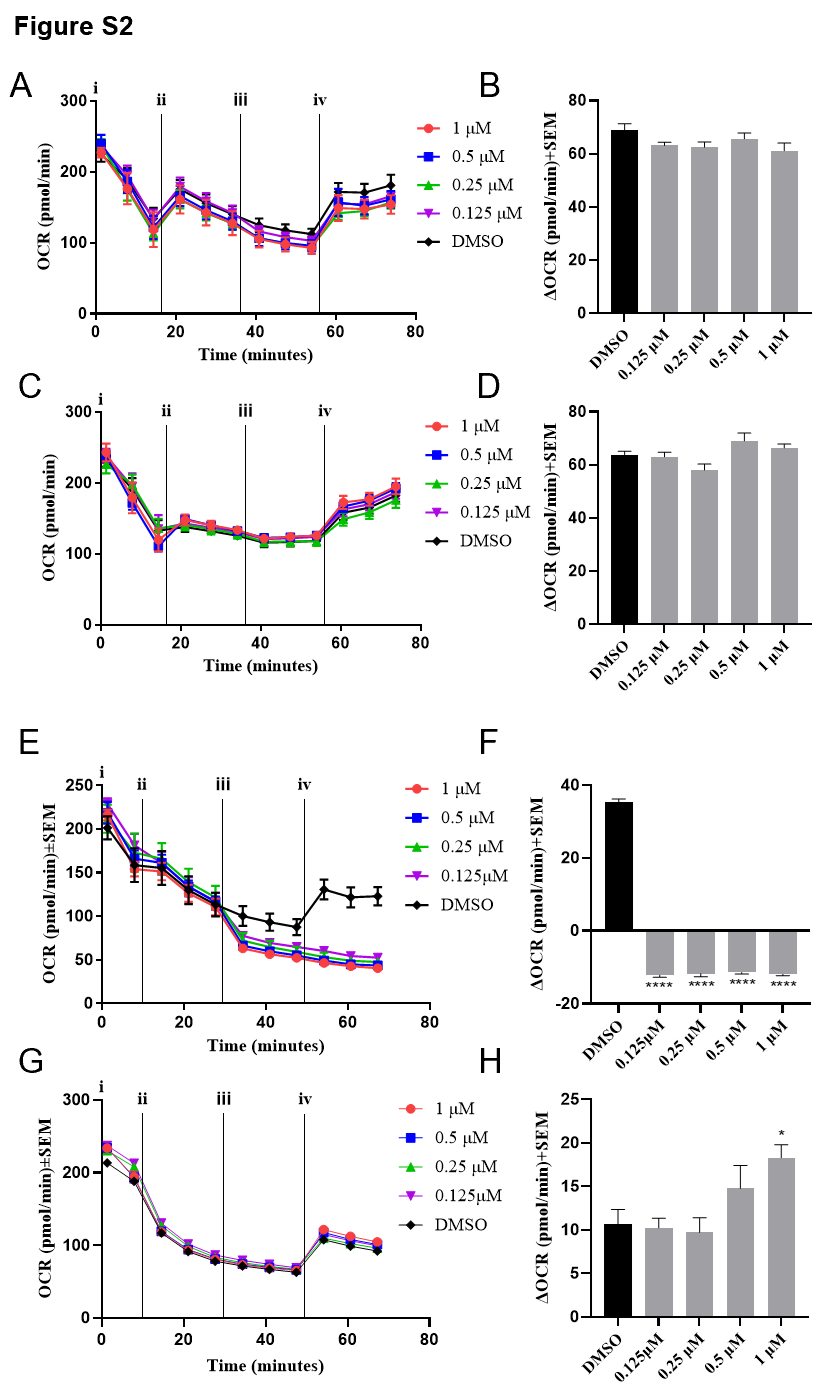


**Figure S2**. (**A-B**) C5 does not alter glutamate driven respiration. (**C-D**) C5 does not alter succinate driven respiration. (**E-H**) Methyl pyruvate reversers C5-inhbited pyruvate driven respiration. All data are representative of the average±SEM of at least three independent experiments. One-way ANOVA analysis was performed to indicate statistical significance between DMSO and compound-treated cultures (*p<0.05, ****p<0.0001).


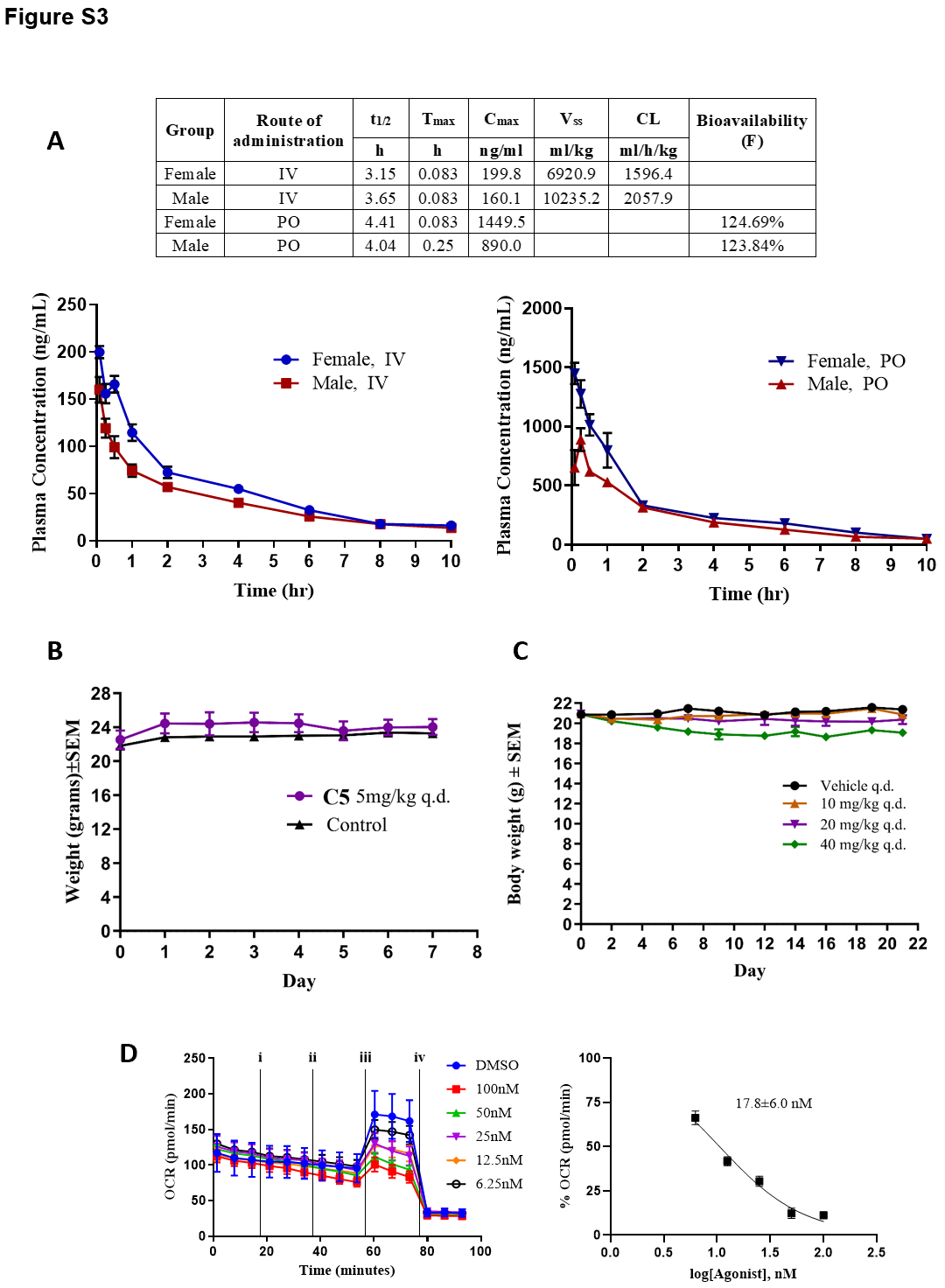


**Figure S3**. (**A**) Pharmacokinetic properties of C5 in male and female mice. MTD studies in healthy mice illustrate that C5 is well tolerated when administered (**B**) intraperitoneally or (**C**) orally. (**D**) C5 inhibits pyruvate driven respiration in permeabilized 67NR cells.

**
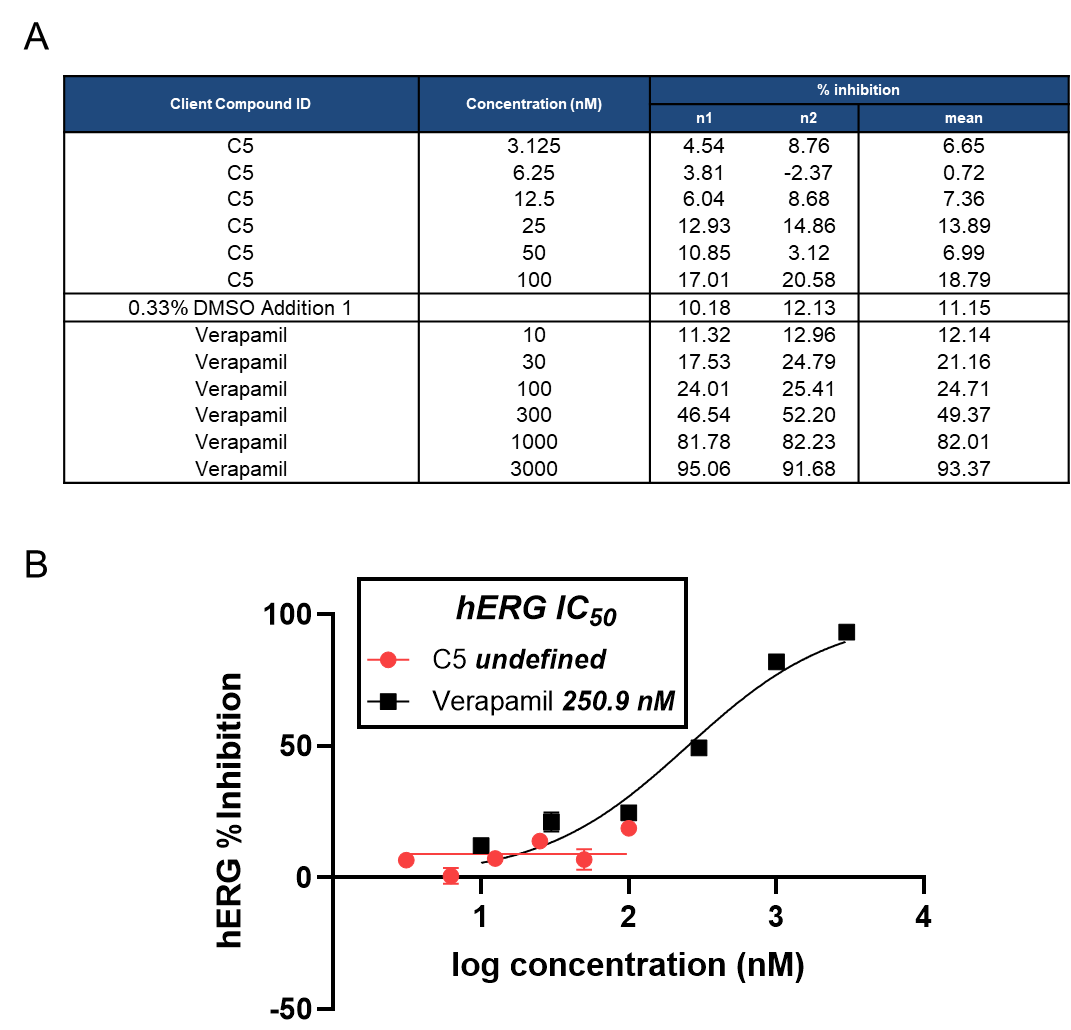
**

**Figure S4.** hERG channel activity studies indicate C5 does not inhibit hERG function. (**A**) Concentrations of **C5** and positive control verapamil used to generate dose-response curves in (**B**). Note concentrations of C5 did not inhibit hERG function, and concentrations selected based on MPC inhibition constant in 4T1 cells.

**Table 1.** Metabolic stability of C3 and C5 in human liver microsomes.

**
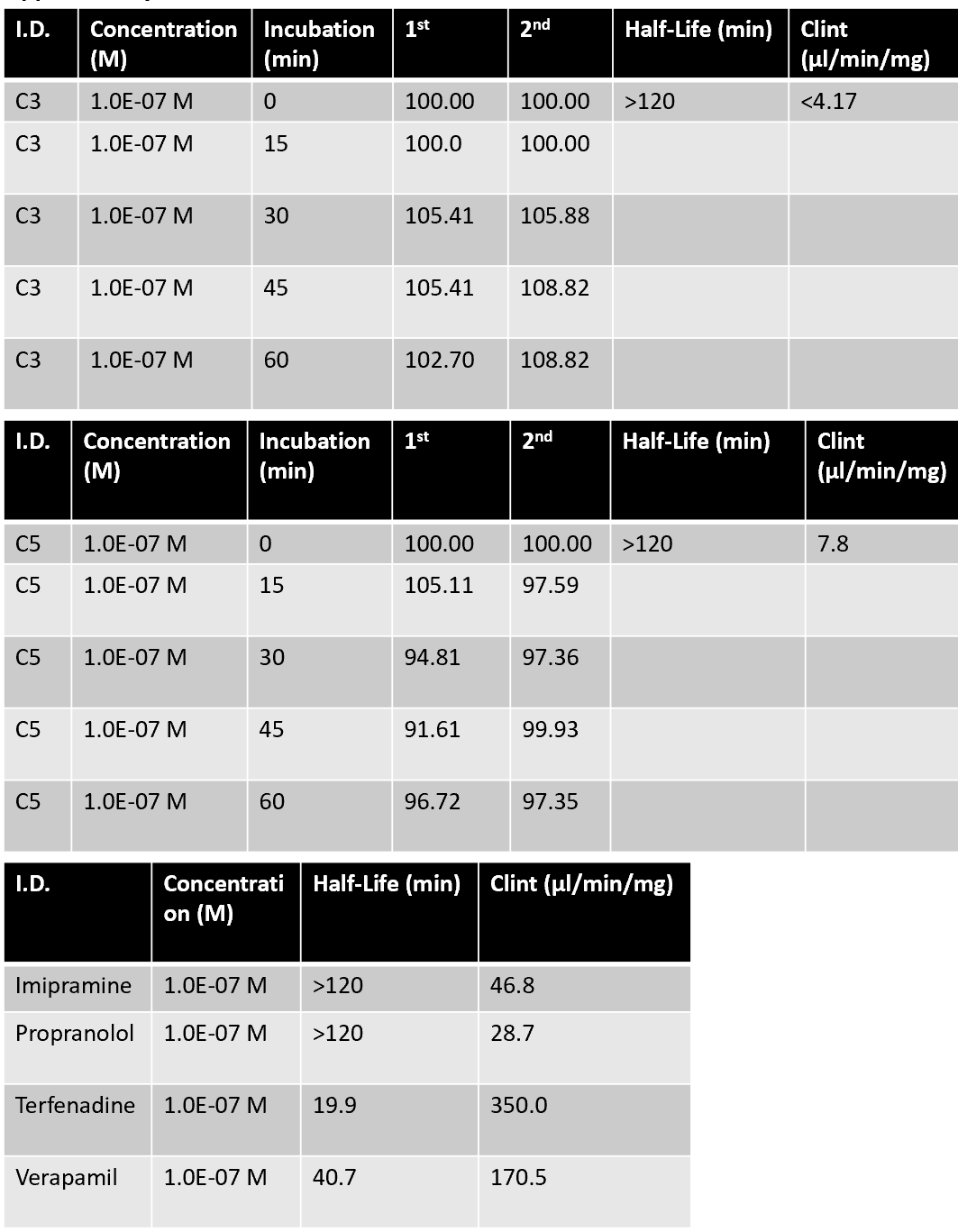
**

**APPENDIX**

***Spectral Characterization***

^1^H- and ^13^C-NMR spectra were plotted on a Bruker-400MHz NMR or Varian-500MHz NMR. High-resolution mass spectra (HRMS) were recorded using a Bruker BioTOF II ESI mass spectrometer.

*7-(bis(4-fluorophenyl)amino)-2-oxo-2H-chromene-3-carboxylic acid* ***(C1)***

**1H NMR (400 MHz, CHLOROFORM-d):** δ 8.72 (s, 1H), 7.45 (d, J= 8.84 Hz, 1H), 7.23-7.20 (m, 4H), 7.15-7.11 (m, 4H), 6.83 (dd, J= 2.28, 8.84 Hz, 1H), 6.70 (d, 2.16 Hz, 1H)

﻿**13C NMR (100 MHz, CHLOROFORM-d):** δ 164.81, 163.63, 161.02 (d, J_CF_ = 246.9 Hz), 156.95, 154.84, 150.44, 140.46 (d, J_CF_ = 3.05 Hz), 131.45, 128.68 (d, J_CF_ = 8.49 Hz), 117.32 (d, J_CF_ = 22.9 Hz), 115.84, 111.42, 109.02, 103.42

**HRMS (ESI) m/z:** calc’d for C_22_H_13_F_2_NO_4_ [M+H^+^]: 394.0885 found: 394.0917

*7-((4-fluorobenzyl)(methyl)amino)-2-oxo-2H-chromene-3-carboxylic acid* ***(C4)***

**1H NMR (400 MHz, CHLOROFORM-d):** δ 12.28 (s, 1H), 8.65 (s, 1H), 7.46 (d, J= 9.00 Hz, 1H), 7.16-7.13 (m, 2H), 7.05 (t, J= 8.52 Hz, 2H), 6.75 (dd, J= 2.28, 8.96 Hz, 1H), 6.58 (d, J= 2.08Hz, 1H), 4.68 (s, 2H), 3.23 (s, 3H).

﻿**13C NMR (100 MHz, CHLOROFORM-d):** δ 165.29, 164.09, 162.31 (d, J_CF_ = 245.19 Hz), 157.64, 155.15, 150.50, 131.88, 131.54 (d, J_CF_ = 3.23 Hz), 127.92 (d, J_CF_ = 8.09 Hz), 116.09 (d, J_CF_ = 21.55 Hz), 111.28, 109.21, 106.85, 97.77, 55.66, 39.31.

**HRMS (ESI) m/z:** calc’d for C_18_H_14_FNO_4_ [M+H^+^]: 328.0980 found: 328.0994

*7-((4-fluorophenyl)(methyl)amino)-2-oxo-2H-chromene-3-carboxylic acid* ***(C2)***

**1H NMR (400 MHz, CHLOROFORM-d):** δ 12.24 (s, 1H), 8.66 (s, 1H), 7.41 (d, J= 8.92 Hz, 1H), 7.27-7.17 (m, 4H), 6.65 (dd, J= 1.4, 8.9 Hz, 1H), 6.57 (s, 1H), 3.43 (s, 3H)

﻿**13C NMR (100 MHz, CHLOROFORM-d):** δ 165.19, 163.98, 161.51 (d, J_CF_ = 246.9 Hz), 157.41, 155.34, 150.53, 141.43 (d, J_CF_ = 3.13 Hz), 131.41, 128.86 (d, J_CF_ = 8.54 Hz), 117.45 (d, J_CF_ = 22.6 Hz), 112.63, 109.71, 107.34, 98.86, 40.84

**HRMS (ESI) m/z:** calc’d for C_17_H_12_FNO_4_ [M+H^+^]: 314.0823 found: 314.0842

*7-(benzyl(methyl)amino)-2-oxo-2H-chromene-3-carboxylic acid* ***(C3)***

**1H NMR (500 MHz, CHLOROFORM-d):** δ 12.32 (s, 1H), 8.65 (s, 1H), 7.45 (d, J= 7.2 Hz, 1H), 7.38-7.27 (m, 3H), 7.17 (d, J= 6.8 Hz, 2H), 6.79 (dd, J= 1.20, 7.20 HZ, 1H), 6.60 (s, 1H), 4.72 (s, 2H), 3.25 (s, 3H).

﻿**13C NMR (100 MHz, CHLOROFORM-d):** δ 165.36, 164.18, 157.66, 155.32, 150.47, 135.84, 131.82, 129.11, 127.83, 126.22, 111.35, 109.12, 106.58, 97.70, 56.31, 39.45.

**HRMS (ESI) m/z:** calc’d for C_18_H_15_NO_4_ [M+H^+^]: 310.1074 found: 310.1083

***Spectral characterization of 1,3-dihydroxy-2-(hydroxymethyl)propan-2-aminium 7-(methyl(4-(trifluoromethyl)benzyl)amino)-2-oxo-2H-chromene-3-carboxylate (C5)***


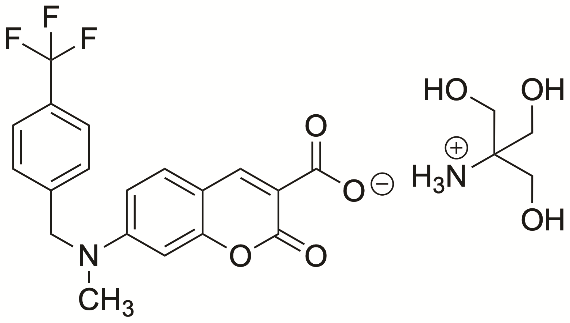


**^1^H NMR (400 MHz, DMSO-d6):** δ 8.209 (s, 1H), 7.6814 (d, 2H, J= 25.92), 7.5125 (d, 1H, J-8.72 Hz), 7.4255 (d, 2H, J= 7.68 Hz), 6.7399 (s, 1H, J= 8.8 Hz), 6.5566 (s, 1H), 4.8303 (s, 2H), 3.1767 (s, 3H)

**^13^C NMR (100 MHz ,DMSO-d6):** δ 167.9491, 159.1321, 156.8593, 152.7093, 144.7865, 143.6035, 130.4881, 128.14085 (q, J= 31.5 Hz), 127.7736, 125.9397 (q, J= 3.6 Hz), 124.7483 (q, J= 270.4 Hz), 119.0451, 109.8017, 108.9669, 97.4376, 60.9012, 60.5096, 55.1307, 39.5101

**^19^F NMR ( 376 MHz DMSO-d6):** δ 60.8502

**HRMS (ESI) m/z:** calculated for C_19_H_14_F_3_NO_4_ [M+1Na]^+^: 400.0767, found 400.0774


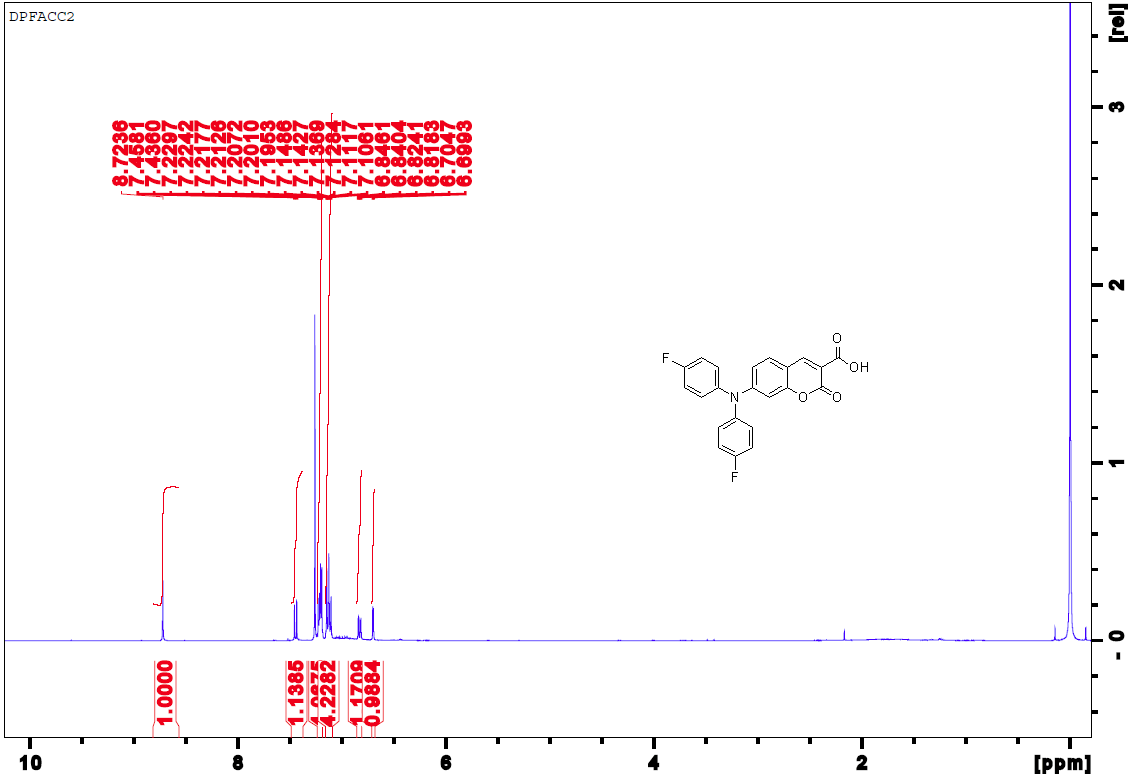


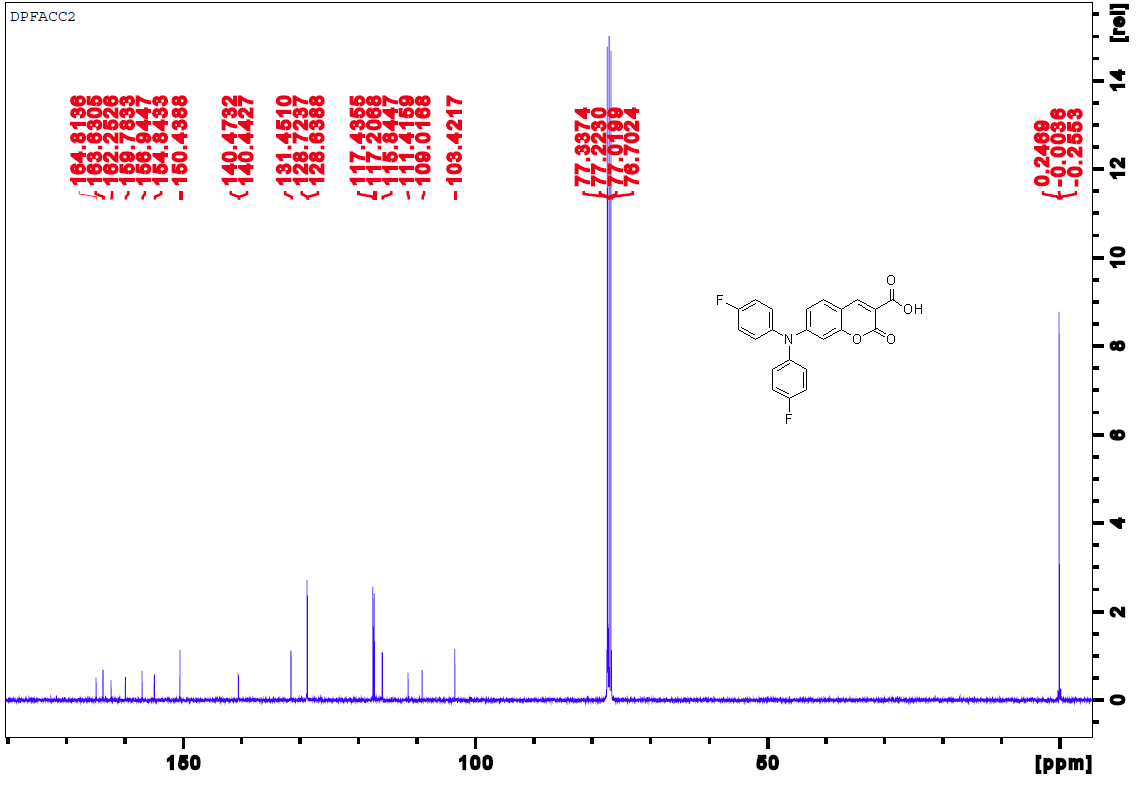


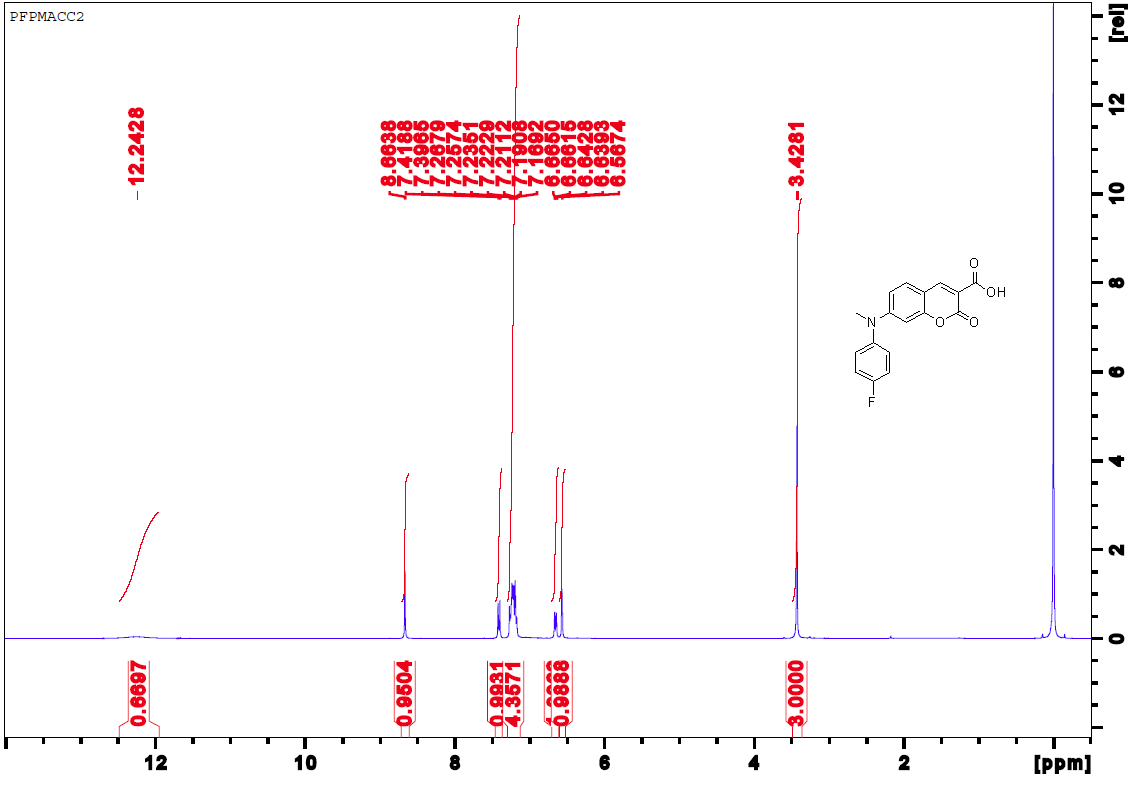


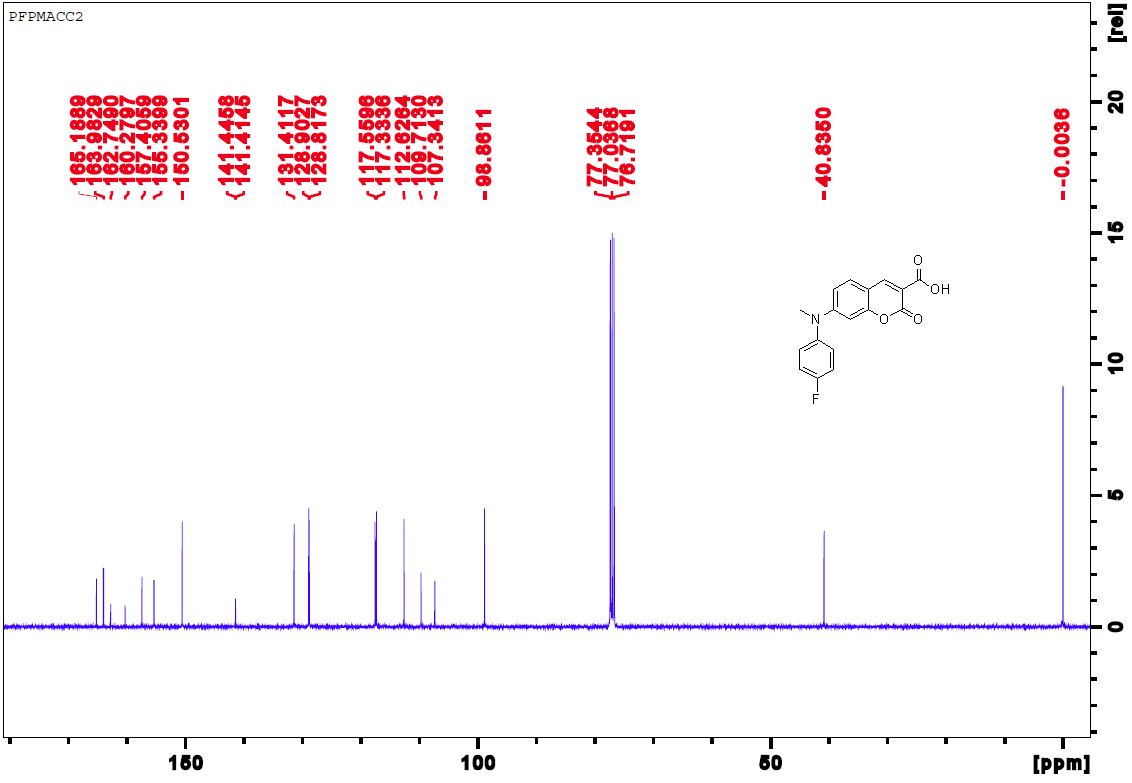


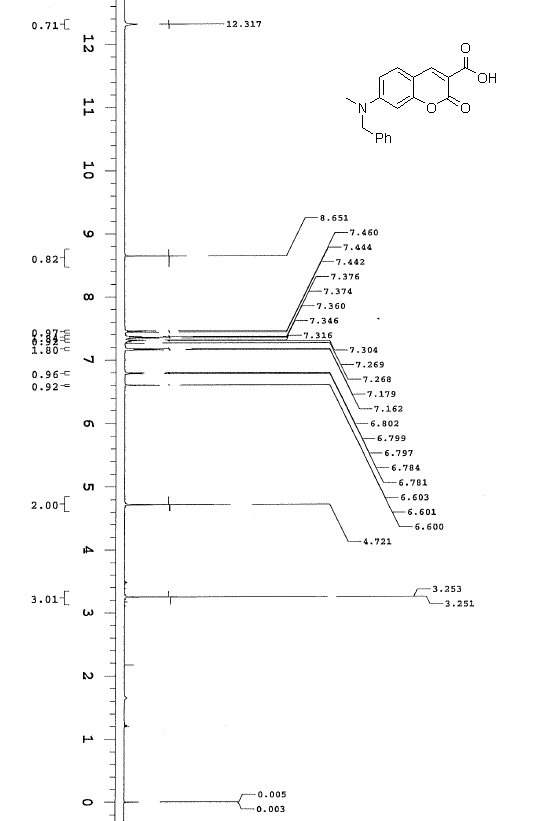


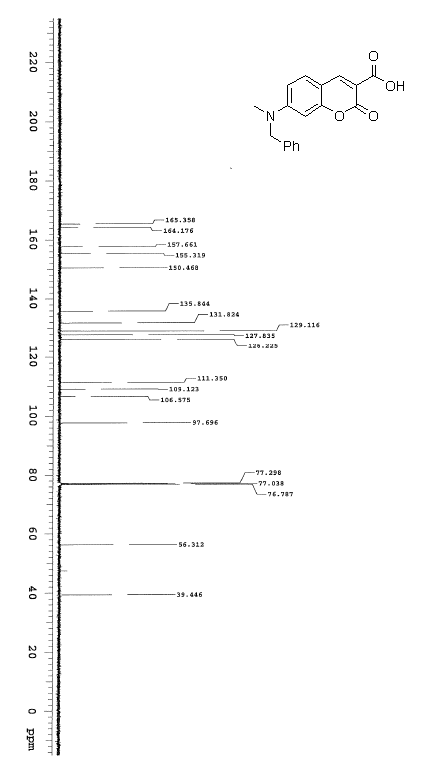


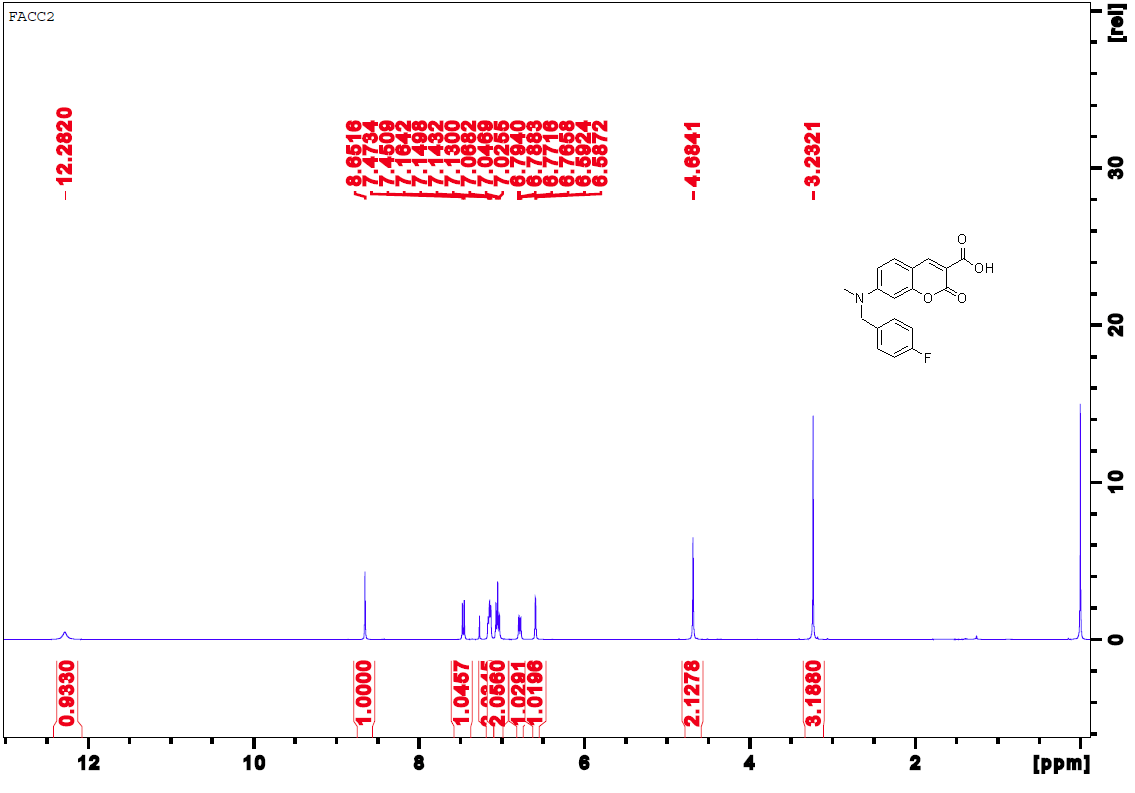


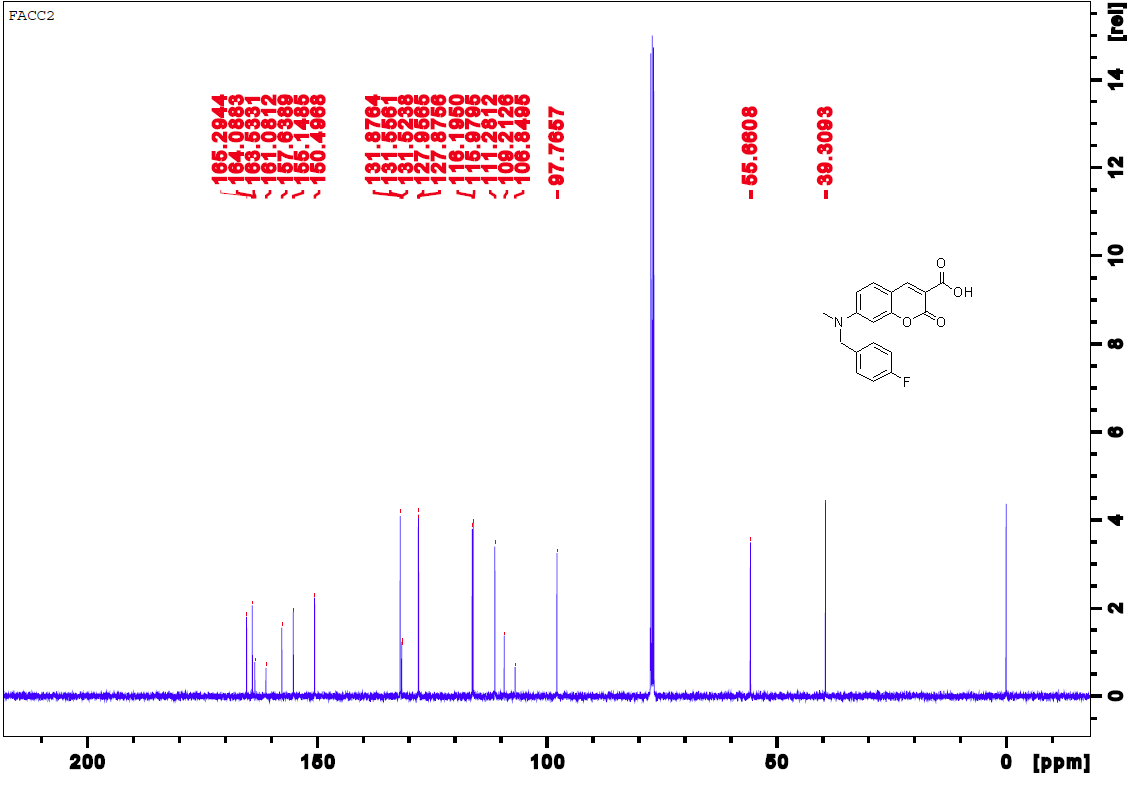


**C5**


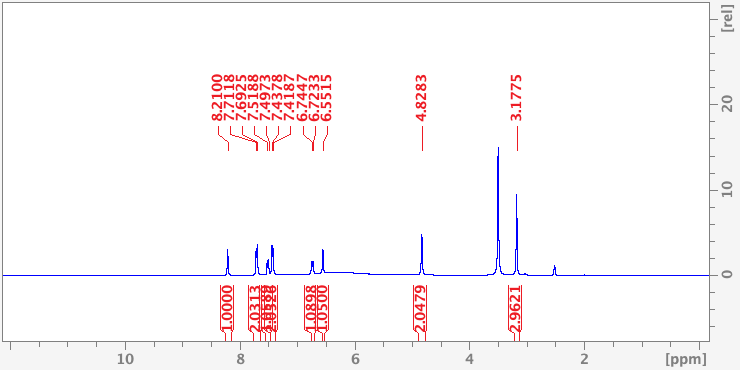


**C5**


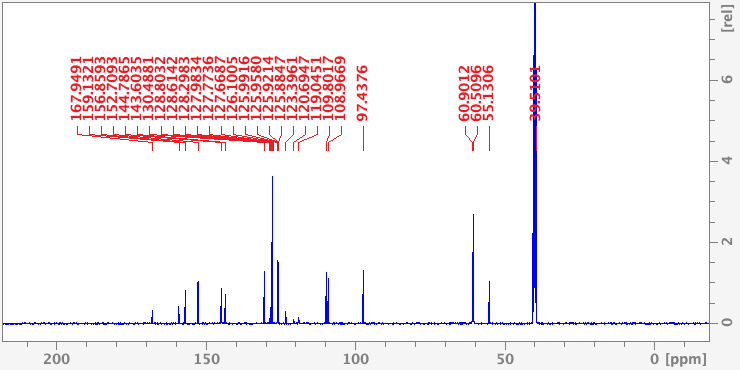

Supplement: Supporting information [file mmc1.docx]
