# Supplementary figures and images for: Development of novel mitochondrial pyruvate carrier inhibitors for breast cancer treatment
Source: J Biol Chem. 2025 Jul 16;301(8):110486. doi: 10.1016/j.jbc.2025.110486 (PMC12355554; doi:10.1016/j.jbc.2025.110486)

Figure 1B

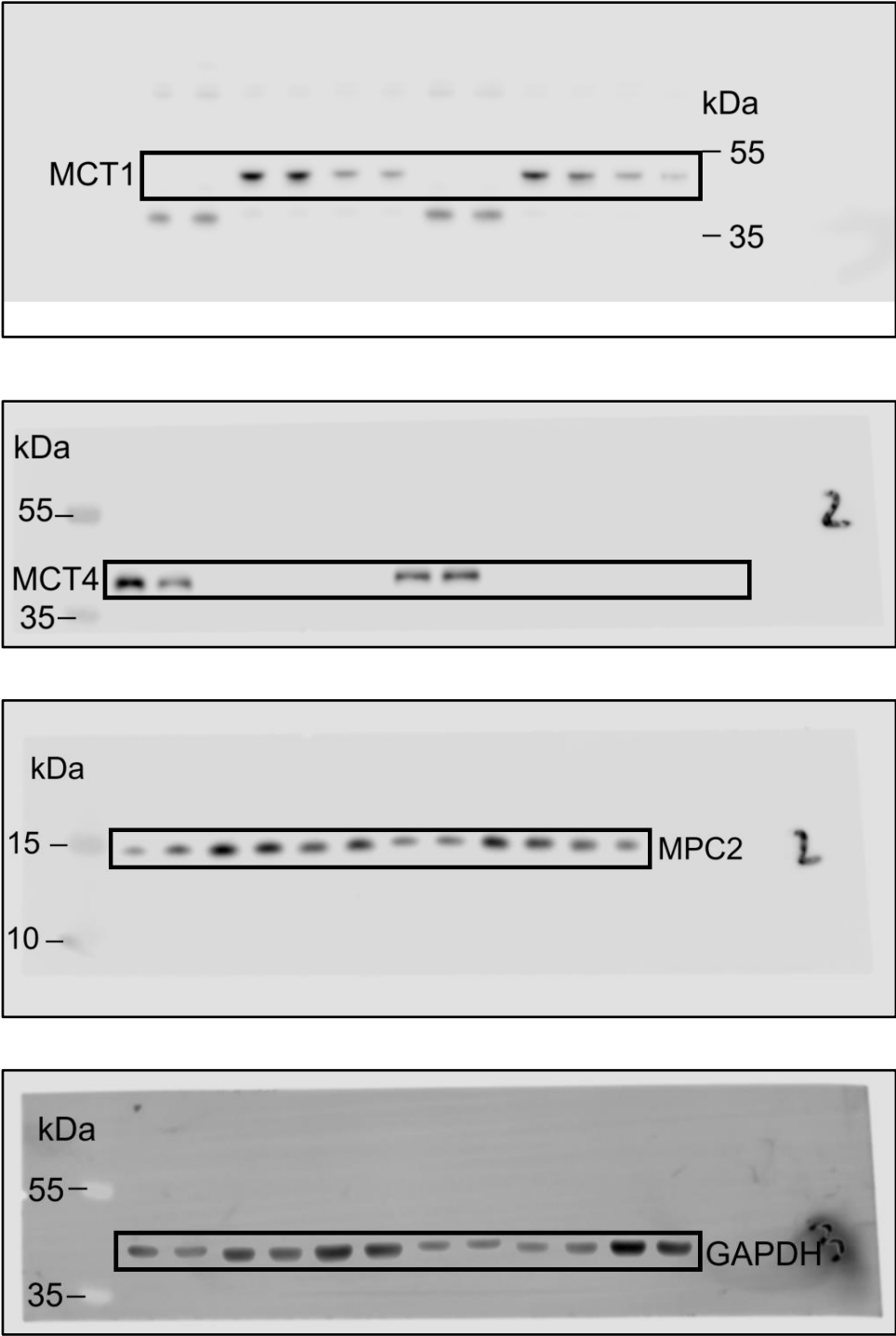

Figure 5A

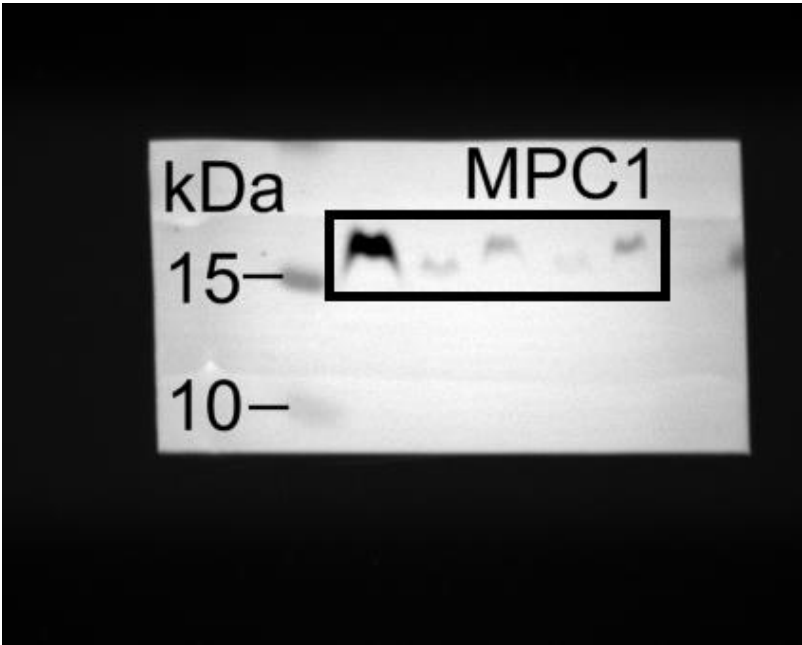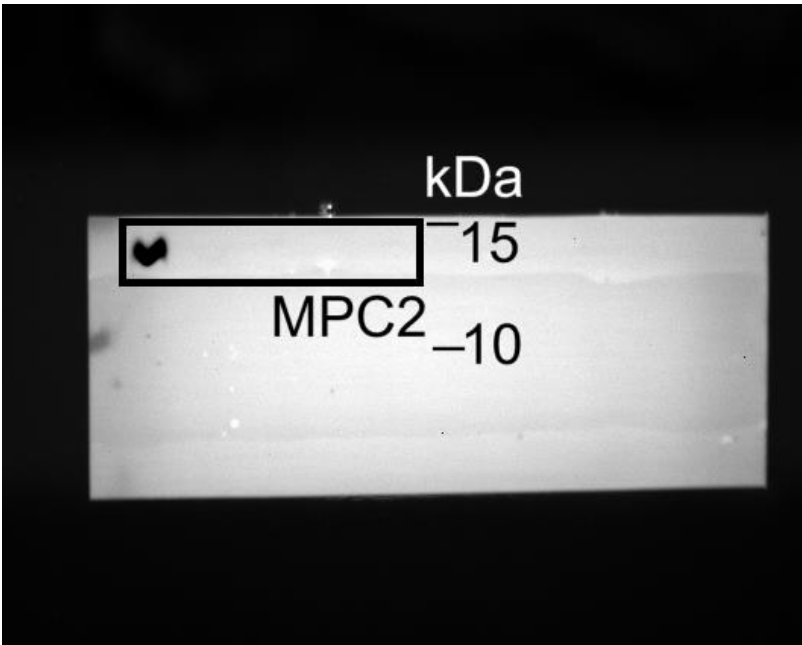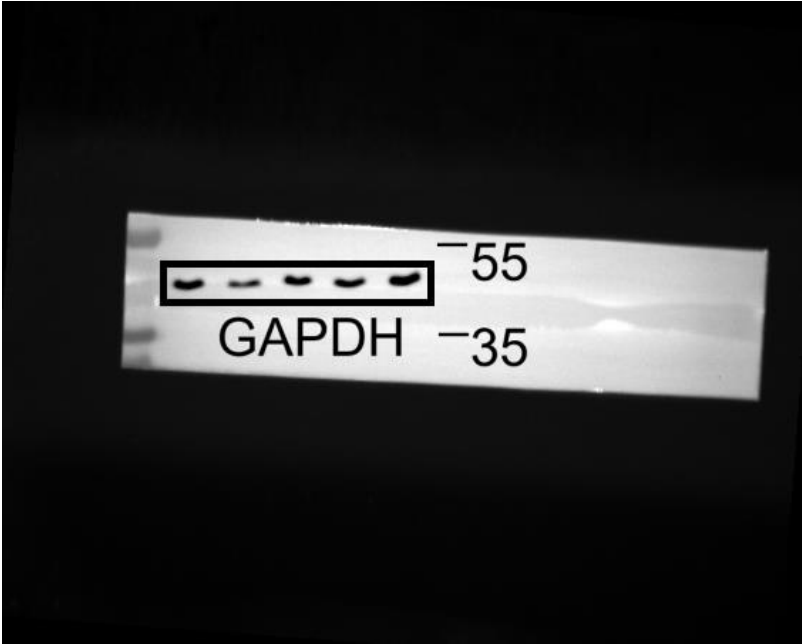

Supplement: Uncropped Blots [file mmc2.pdf]
